# Bisection of the X chromosome disrupts the initiation of chromosome silencing during meiosis in *Caenorhabditis elegans*

**DOI:** 10.1038/s41467-021-24815-0

**Published:** 2021-08-10

**Authors:** Yisrael Rappaport, Hanna Achache, Roni Falk, Omer Murik, Oren Ram, Yonatan B. Tzur

**Affiliations:** 1 Department of Genetics, The Institute of Life Sciences, The Hebrew University of Jerusalem, Jerusalem, Israel; 2 Medical Genetics Institute, Shaare Zedek Medical Center, Jerusalem, Israel; 3 Department of Biological Chemistry, The Institute of Life Sciences, The Hebrew University of Jerusalem, Jerusalem, Israel

**Keywords:** Chromosomes, Germline development, CRISPR-Cas9 genome editing, Development

## Abstract

During meiosis, gene expression is silenced in aberrantly unsynapsed chromatin and in heterogametic sex chromosomes. Initiation of sex chromosome silencing is disrupted in meiocytes with sex chromosome-autosome translocations. To determine whether this is due to aberrant synapsis or loss of continuity of sex chromosomes, we engineered *Caenorhabditis elegans* nematodes with non-translocated, bisected X chromosomes. In early meiocytes of mutant males and hermaphrodites, X segments are enriched with euchromatin assembly markers and active RNA polymerase II staining, indicating active transcription. Analysis of RNA-seq data showed that genes from the X chromosome are upregulated in gonads of mutant worms. Contrary to previous models, which predicted that any unsynapsed chromatin is silenced during meiosis, our data indicate that unsynapsed X segments are transcribed. Therefore, our results suggest that sex chromosome chromatin has a unique character that facilitates its meiotic expression when its continuity is lost, regardless of whether or not it is synapsed.

## Abstract (web summary)

During meiosis, sex chromosomes show silenced gene expression that is disrupted when translocated to autosomes. Here, the authors engineer *C. elegans* with bisected X chromosomes and see active transcription in gonads, indicating continuity of sex chromosomes promotes silencing.

## Introduction

During prophase I of meiosis in most sexually reproducing organisms, homologous chromosomes pair and then undergo a close engagement known as synapsis to complete interhomolog crossover recombination^1–12^. In the heterogametic cells of many species (e.g., meiocytes with X and Y chromosomes), the sex chromosomes pair but undergo synapsis and crossovers only in the pseudo-homology regions. In mouse testes, these unsynapsed chromosomes form a compartment of heterochromatic chromatin referred to as the XY body, which undergoes transcriptional silencing through many stages of meiosis and, in some cases, into gametogenesis. Although sex chromosomes have appeared and disappeared several times during metazoan evolution, meiotic sex chromosome inactivation (MSCI) occurs in many species from worms to humans^13–17^.

Chromosome silencing in mammalian spermatogenesis is perturbed by mutations in genes involved in meiotic double-strand break formation, DNA damage response, and chromatin modification^18–23^. In mouse testes, the lack of MSCI usually leads to pachytene arrest, apoptosis, and persistence of homologous recombination intermediates^24,25^. Although the molecular mechanism of the silencing emplacement is well characterized, our knowledge of how it is triggered is lacking.

In *C. elegans* gonads, nuclei are arranged according to developmental progression. At the distal end, proliferative cells undergo mitotic divisions, and they enter meiosis at the leptotene/zygotene stage, where homologous chromosomes pair. Pairing is closely followed by synapsis within an evolutionarily conserved structure involving lateral and central proteinaceous elements that keep the homologs aligned. The chromosomes fully synapse during pachytene, which allows crossovers to mature. In hermaphrodite worms, the nuclei proceed through diplotene and reach maturity at the diakinesis stage^7,8,12^. In XO male worms, the single X chromosome does not undergo synapsis and is transcriptionally silenced throughout meiosis^26–29^. In hermaphrodites, the two X chromosomes pair and synapse, yet these chromosomes are only transiently silenced in early meiotic stages. Toward the end of pachytene the silencing is relieved, however, and transcription from these chromosomes increases. This transient silencing was found to be important for normal germline development and regulated by the *mes* genes^28,30^.

The current model views sex chromosome silencing in heterogametic meiocytes as a special case of meiotic silencing of unsynapsed chromatin (MSUC)^31^, a process characterized in mammals, *Neurospora crassa* and *C. elegans*^31–33^. Several lines of evidence support this model including the silencing of the unsynapsed X chromosome in XO female mouse meiocytes^31^ and the lack of silencing in synapsed Y chromosomes in mouse XYY testes^34^. Furthermore, when translocations between autosomes and sex chromosomes occur, the localization of MSCI effectors to the sex chromosomes segments is perturbed^35–38^. The lack of chromosome silencing has been explained by the aberrant synapsis of the sex chromosome segments observed in these nuclei. Nevertheless, in some cases, silencing markers are detected on synapsed, translocated sex chromosomes^35–38^, raising the possibility that changes in sex chromosome continuity can also perturb chromosome silencing.

In this study, we tested the hypothesis that sex chromosomes must be continuous and undivided for efficient silencing during meiosis. We created stable worm strains with bisected X chromosomes that did not translocate to autosomes. We found that in meiocytes of these strains, and in a strain with a reciprocal translocation of chromosomes V and X, the transient silencing of the X in hermaphrodites was reduced. The X chromosome segments of both males and hermaphrodites stained more strongly with markers associated with transcription in mutant than in wild-type worms, and the expression of X-linked genes in the gonads was increased in the mutants. In contrast to the prediction that silencing of sex chromosomes in meiosis is a special case of MSUC, we showed that segments of the X that are unsynapsed are not silenced. Based on these data, we suggest that chromosome continuity is required in *C. elegans* for X chromosome silencing and proper meiotic progression.

## Results

### Creation of stable, homozygous, *C. elegans* strain with bisected X chromosomes

Previous reports indicated that meiotic silencing in sex chromosomes is disrupted in heterogametic cells with translocations between sex chromosomes and autosomes and that, in some cases, gene expression was uncoupled from the synapsis state of the translocated chromosomes^31,35,36,38,39^. This suggested that disruption of chromosome continuity prevented initiation of the sex chromosome silencing. To test this hypothesis, we aimed to create worm strains with disruptions in X chromosome continuity but without a translocation with an autosome, reducing the possibility of aberrant synapsis. Ideally, we wanted a system that (1) is homozygous stable, (2) has segments considerably smaller than the full-size chromosome but larger than extra-chromosomal arrays and free duplications, and (3) has segments with telomeres on both ends.

Previous reports indicated that multiple CRISPR-mediated DNA double-strand breaks at homologous chromosomal loci can lead to chromosomal aberrations such as inversions, large deletions, circularizations, and chromosomal cleavages^40–46^. To create strains with segmented X chromosomes, we searched for genomic regions near the ends of chromosome X with homology to regions at the center. If breaks at both the center and one of the ends are formed and not repaired, three segments are created. If two nonadjacent breaks are ligated, two segments result. If all three segments are ligated, then chromosome rearrangements may occur. A segment without telomers could also undergo circularization as was detected before^40,47^. We identified a 2.2-kb region (X:16508962-16511217) on the right side of the X chromosome encompassing the noncoding gene *linc-20*, which is homologous (>92% identity) to a region near the center of the X chromosome (X:7769295-7771552) within the 14th intron (i14) of *deg-1*. Neither of these genes have previously been associated with germline roles^48–50^. As previously described^51,52^, we directed Cas9 to these loci with four guide RNAs (gRNAs) to create multiple breaks. We assayed the progeny of worms injected with plasmids for expression of Cas9 and gRNA for deletions in targeted loci using PCR and isolated a strain with small deletions in both loci: The deletion in i14 was 2597 bases, and two deletions were observed in *linc-20* of 1417 and 2721 bases. After five outcrosses with the wild-type strain, the YBT7 strain was established. All further experiments were conducted using this outcrossed strain. This strain was maintained through multiple generations without any change in genotyping markers of these loci.

**Fig. 1:**
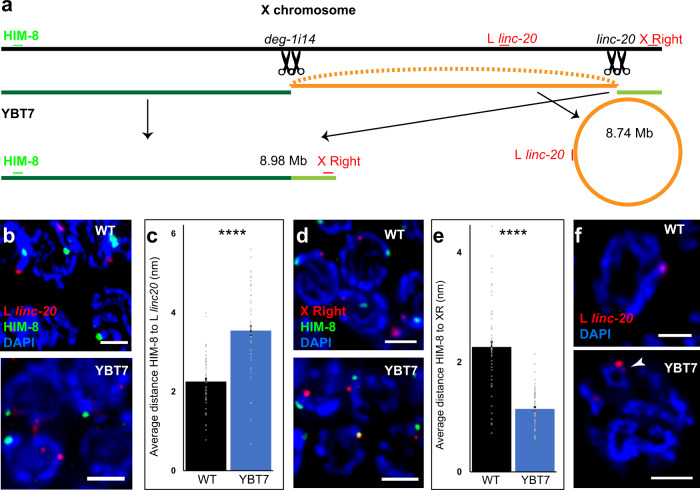
Engineering of worm strains with bisected X chromosomes. **a** Illustration of the X chromosome in a wild-type worm and the segments resulting from Cas9-mediated cleavage during generation of YBT7 worms. The gRNA binding sites (black scissors) and cytological markers (green and red) used in analyses of X chromosome segmentation are indicated. **b** Pachytene nuclei stained with DAPI (blue), anti-HIM-8 (green), and FISH probe complementary to a site to the left of *linc-20* (red). **c** Averages ± SEM of the distance between HIM-8 and the site left of *linc-20*. **d** Pachytene nuclei stained with DAPI (blue), anti-HIM-8 (green), and FISH probe complementary to the right end of the X chromosome (red). **e** Averages ± SEM of the distance between HIM-8 and the site on the right end of chromosome X. **f** Late pachytene nuclei stained with DAPI (blue) and the FISH probe complementary to the site left of *linc-20* (red). The FISH signal associated with the internal segment is marked with an arrowhead. *n* = 80 nuclei. ****p < 0.0001, by the two-tailed Mann–Whitney test. Scale bars = 3 μM.

We next evaluated whether there are structural alterations in the X chromosomes of YBT7 worms using Nanopore long-read DNA sequencing. This analysis indicated that Cas9-mediated cleavages in i14 of *deg-1* and *linc-20* loci resulted in a fusion of the internal segment from X:772344 to X:16511091, ~8.7 Mbp (hence internal segment). The lack of any end in the Nanopore data and cytology evidence suggested that this segment was a circle (Fig. S1a). The Nanopore data further showed that the left fragment was ligated to the right fragment (linking X:7769697 to X:16513803), creating an ~9-Mbp segment (Fig. 1a, referred to as the linear segment). We also detected a small inversion within the fusion point of the linear chromosome (X:7762996 to X:16513802). Sanger sequencing confirmed the fusion points of these segments. No other major chromosomal alterations were detected by Nanopore sequencing.

We verified that both the X chromosomes were segmented in YBT7 by co-staining of YBT7 gonads with antibodies against HIM-8, a protein that binds the left end of the X chromosome^53^, and with fluorescent in situ hybridization (FISH) probes directed to a site to the left of *linc-20* locus (L *linc-20*). Based on Nanopore data, we predicted that in YBT7, HIM-8 should bind to the linear segment, whereas the FISH probes should bind to the internal segment (Fig. 1a). In wild-type pachytene nuclei these markers appeared on the same DAPI-stained track, but in YBT7 the HIM-8 and FISH staining mostly marked different DAPI tracks (Fig. 1b; 80/80 vs. 10/80 on the same track, respectively). Due to the spatial resolution of our fluorescent microscopy two very close tracks are not always differentiated, which is likely why the two markers scored on the same track in a fraction of YBT7 nuclei examined. The distance between the markers was also shorter in wild-type worms than in YBT7 worms (Fig. 1c; 2.25 ± 0.08 vs. 3.5 ± 0.1 μM, respectively, *n* = 80). We next co-stained YBT7 gonads with HIM-8 antibodies and FISH probes directed to the right side of the chromosome (Fig. 1a). The two markers were on the same DAPI-stained track during pachytene in both strains, but in YBT7 they were closer than in the wild-type strain (Fig. 1d, e; 1.1 ± 0.04 vs. 2.3 ± 0.1 μM, respectively, *n* = 80), confirming the segmentation of the X chromosome in YBT7.

Circular chromosomes and large extrachromosomal circular DNA are observed in many organisms in normal and tumor cells and circular chromosomes can be maintained through multiple mitotic divisions^54,55^. In humans, these chromosomal aberrations are thought to result from two double-stranded breaks^47^. Further support for the possibility that the internal segment in YBT7 worms is circular came from imaging of pachytene nuclei marked with a FISH probe designed to hybridize to the internal segment. In YBT7, but not in wild-type gonads, we detected nuclei in which this probe was localized to a circular DAPI-stained track (Figs. 1f and S1b). Taken together, these analyses indicate that YBT7 worm cells have a stably segmented X chromosome. These worms are homozygous for two dissociated parts of the X chromosome that are not translocated to autosomes.

### Silencing markers are lost in early meiotic nuclei with bisected X chromosomes

**Fig. 2:**
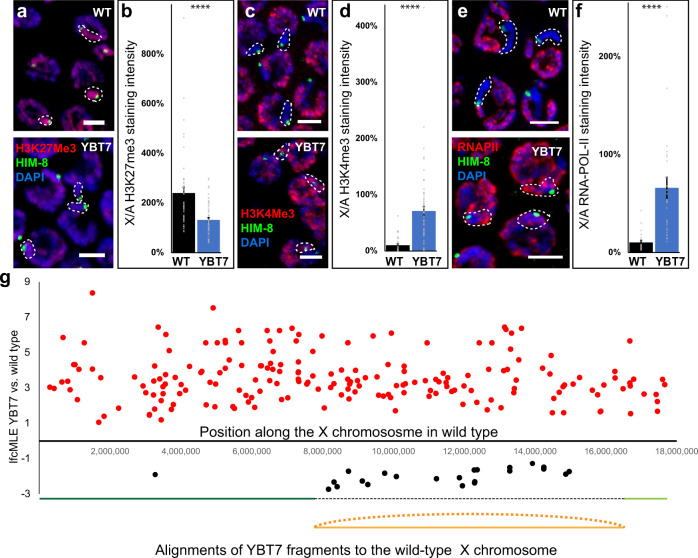
Transcription of X segments is higher in meiocytes of YBT7 than wild type. **a** Mid-pachytene nuclei stained with DAPI (blue), anti-HIM-8 (green), and antibody against H3K27me3 (red). **b** Averages ± SEM of the relative H3K27me3 signals in wild-type and YBT7 nuclei on HIM-8-marked body vs. an autosome. *n* = 47 and 55 nuclei for WT and YBT7, respectively. **c** Mid-pachytene nuclei stained with DAPI (blue), anti-HIM-8 (green), and antibody against H3K4me3 (red). **d** Averages ± SEM of the relative H3K4me3 signals in wild-type and YBT7 nuclei on HIM-8-marked body vs. an autosome. *n* = 33 and 66 nuclei for WT and YBT7, respectively. **e** Mid-pachytene nuclei stained with DAPI (blue), anti-HIM-8 (green), and antibody against RNAPII (red). **f** Averages ± SEM of the relative RNAPII signals in wild-type and YBT7 nuclei on HIM-8-marked body vs. an autosome. *n* = 22 and 31 nuclei for WT and YBT7, respectively. ****p < 0.0001, by the two-tailed Mann–Whitney test. Scale bar = 3 μM. **g** RNA-seq results. Plotted is log_2_ of the fold-change maximum likelihood estimate (lfcMLE) of YBT7 vs. wild type for each gene along the X chromosome. Only highly differentially expressed genes are illustrated. Alignments of the linear (green) and internal (orange) segments of YBT7 are shown schematically below the graph.

In the gonads of hermaphroditic *C. elegans*, the two X chromosomes are silenced from the proliferative nuclei until late pachytene, and then transcription resumes^26–29,56^. During early meiotic stages, the chromatin of X chromosomes is enriched with modifications associated with heterochromatin assemblies that correlate with low transcriptional activity such as histone H3 trimethylated at lysine 27 (H3K27me3)^57^. We tested whether bisection of the X chromosome changed the chromatin state by staining the gonads with anti-H3K27me3 antibodies and with anti-HIM-8 to mark the X chromosome. As shown previously^57^, we found that in early wild-type pachytene nuclei the X chromosomes were strongly stained with H3K27me3 antibodies (Fig. 2a). In YBT7 mid-pachytene nuclei, the HIM-8-marked DAPI track was stained with the H3K27me3 antibody at levels similar to all other DAPI tracks, and no chromosome was strongly stained (Fig. 2a). We next measured the level of H3K27me3 signal associated with the HIM-8-marked chromosome relative to the level associated with the autosomes in the same nucleus. We found that in the wild-type strain the ratio was 2.4 ± 0.2, whereas that in the YBT7 strain was 1.3 ± 0.09 (Fig. 2b; *n* ≥ 47, *p* < 0.0001 by the Mann–Whitney test). Thus, the linear segment of the X chromosome in early meiotic YBT7 nuclei was marked by H3K27me3 at levels closer to autosomes.

We next tested whether transcription from the X chromosome changes when it is bisected. For this analysis, we used antibodies to histone H3 trimethylated at lysine 4 (H3K4me3), a modification associated with euchromatin assembly that correlates with active transcription in *C. elegans*^58^. In nuclei in early meiotic stages in wild-type gonads, there are very low levels of H3K4me3 on the X chromosomes^26,57–59^. In YBT7 nuclei, however, the X chromosome segment was more strongly stained than in wild-type gonads; the level was similar to that of autosomes (Fig. 2c). Quantification of the staining on the HIM-8-marked chromosome vs. the autosomes within the same nucleus indicated that the ratio was significantly higher in YBT7 nuclei than in wild-type nuclei (Fig. 2d; 0.10 ± 0.02 vs. 0.71 ± 0.08, respectively, *n* ≥ 33, *p* < 0.0001 by the Mann–Whitney test). The absolute fluorescence signal from both H3K27me3 and H3K4me3 on the autosomes was not significantly different between wild-type and YBT7 nuclei (Fig. S2), suggesting that the difference does not result from a general change in transcription. In contrast to wild type, staining of the X segments in YBT7 with H4K20me and H3K36me3 antibodies, which mark heterochromatin and euchromatin assemblies^60–62^, respectively, was similar to that on autosomes. This further supports the indications for increased transcription from these segments (Fig. S3).

One of the most direct cytology markers of active transcription is the antibody that recognizes the B1 subunit of RNA polymerase II (RNAPII) when phosphorylated at Ser2^26,63,64^. In agreement with previous observations^26^, we found that in mid-pachytene nuclei of wild-type gonads the X chromosome was not strongly associated with this antibody (Fig. 2e). In contrast, in YBT7 mid-pachytene nuclei, the chromatin tracks stained with anti-HIM-8, indicative of the X chromosome segment, were strongly stained for active RNAPII (Fig. 2e). The ratio of RNAPII signal on the HIM-8-associated chromosome vs. an autosome within the same nucleus was dramatically different between wild-type and YBT7 gonads (Fig. 2f; 0.10 ± 0.02 vs. 0.7 ± 0.1, respectively, *n* ≥ 22, *p* < 0.0001 by the Mann–Whitney test).

**Fig. 3:**
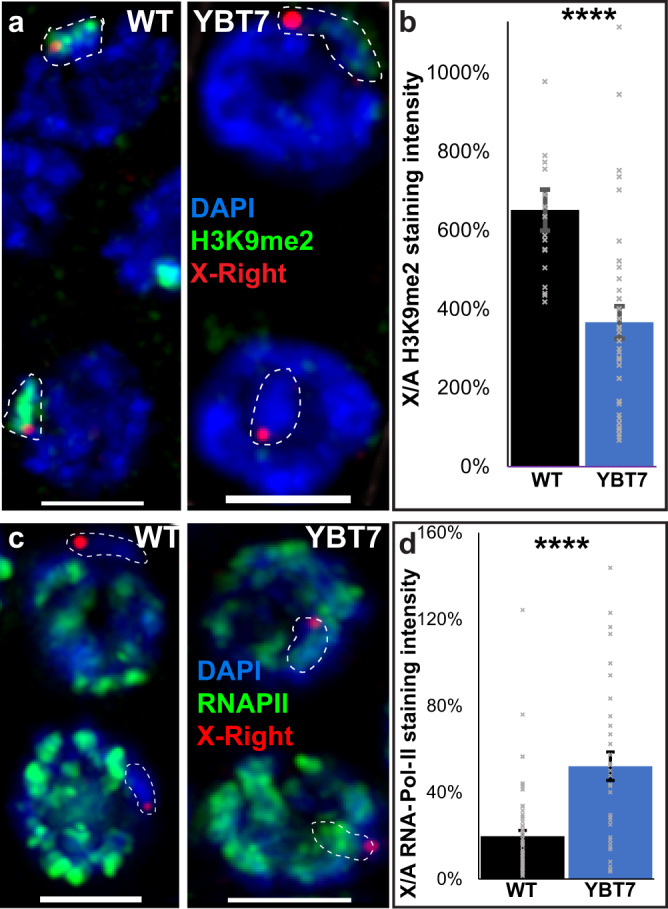
Bisected X segments are stained with markers associated with transcription during male meiosis. **a** Mid-pachytene male nuclei stained with DAPI (blue), X-right FISH (red), and antibody against H3K9me2 (green). **b** Averages ± SEM of the relative H3K9me2 signals in wild-type and YBT7 nuclei on HIM-8-marked body vs. an autosome. *n* = 17 and 36 nuclei for WT and YBT7 respectively. *p* value = 0.00003 by the two-tailed Mann–Whitney test. **c** Mid-pachytene male nuclei stained with DAPI (blue), X-right FISH (red), and antibody against RNAPII (green). **d** Averages ± SEM of the relative RNAPII signals in wild-type and YBT7 nuclei on HIM-8-marked body vs. an autosome. *p* value = 0.000004, by the two-tailed Mann–Whitney test. *N* = 62 and 34 nuclei for WT and YBT7 respectively. ****p < 0.0001, by the two-tailed Mann–Whitney test. Scale bar = 3 μM.

We next examined male gonads in which the single unsynapsed X chromosome is silenced throughout spermatogenesis. In wild-type mid-pachytene nuclei the X chromosome was strongly stained with antibodies against H3K9me2, a modification associated with heterochromatin assemblies which correlates with low transcriptional activity^26^. In YBT7 male gonads the difference between the staining on the segment associated with the X-right FISH mark and the autosomes was significantly decreased relative to that in wild-type gonads (Fig. 3a, b; *n* ≥ 17, *p* < 0.0001 by the Mann–Whitney test). The opposite was found when we tested RNAPII. In wild-type mid-pachytene nuclei, the autosomes were stained at much higher levels with RNAPII compared to the X-right-marked body, and the difference was significantly smaller in YBT7 gonads (Fig. 3c, d; *n* ≥ 34, *p* < 0.00001 by the Mann–Whitney test). We noted that in males the change in the relative staining between the X and the autosomes in YBT7 was smaller compared to hermaphrodites. We speculate that this stems from the 2X:2A ratio of DAPI-stained tracks in hermaphrodite vs. the 1X:2A in males, yet we cannot rule out the involvement of other molecular processes which are different between males and hermaphrodites. Taken together these results indicate that in YBT7 nuclei, the linear segment of the bisected X chromosome is not silenced during early meiotic steps.

### Many X-linked genes are upregulated in YBT7 gonads

Our cytological data suggest that the X chromosome linear segment in YBT7 gonads does not undergo meiotic silencing. To determine whether specific regions of the X segments are transcriptionally silent, we dissected gonads from wild-type and YBT7 worms and compared their transcriptomes. We found that 197 genes of 2867 from the X chromosome were highly upregulated and 24 were highly downregulated in YBT7 compared to wild-type gonads (Supplementary Data 1). Of the highly upregulated genes, 91 are encoded on the internal segment and 106 on the linear segment, and no specific regions were over- or underrepresented (Fig. 2g, Supplementary Data 1). This is probably an underestimation of the level of upregulated genes since the loss of X chromosome silencing in hermaphrodites is expected to affect expression only at the distal part of the gonads, whereas we sequenced RNA from whole gonads. Moreover, mRNA abundance is higher at the proximal than in the distal side of the gonad^65^. The number of upregulated genes is comparable to numbers reported from studies of the germlines of mutant worms defective in the transient silencing of the X-chromosome. For example, Bender et al. identified 61 X-linked genes upregulated in *mes-4* mutants^28^ and Gaydos et al. identified 154 genes^30^. Similarly, in *mes-2* mutants, only 16 X-linked genes were found to be upregulated^30^.

Among the 24 highly downregulated genes in YBT7 gonads, only two were on the linear segment, and 22 were on the internal segment (Fig. 2g and Supplementary Data 1, *p* < 0.01 by Fisher’s exact test). It was surprising that the downregulated genes were not evenly distributed between segments, although given the limited number of downregulated genes, which were far fewer than upregulated genes, the difference may not be significant. The discrepancy between numbers of downregulated genes on the linear and internal segments could stem from the loss of the internal segment in some meiocytes or from a complex genetic plan that influences these downregulated genes. To test the former possibility, we quantified FISH probes directed to the internal segment in mid-pachytene nuclei and observed one or two foci per nucleus in YBT7. We note that this analysis could be skewed due to synapsis (Fig. S4, see below). We also sequenced the genomes of the parental wild-type strain and YBT7 at ~100X coverage using Illumina next-generation sequencing but did not find any major changes in copy number between the bisected segments. These data suggest that no major copy number loss occurred in the internal segment, and thus it is likely that the downregulated genes are the result of the activated expression of other genes from the X segments (see discussion).

The dramatic difference we found in the expression of genes on the X chromosome in YBT7 gonads compared to wild-type gonads could be correlated with differences in autosomal transcription. Indeed, the abundances of 706 autosomal genes were also higher in YBT7 gonads than wild-type gonads (Fig. S5 and Supplementary Data 1). Nevertheless, a higher percentage of genes were upregulated on the segmented X chromosome than on autosomes (*p* < 7.29 × 10^−17^ by the hypergeometric test). The loss of silencing of X-linked genes may lead to misregulation of autosomal gene expression. However, a specific genetic program was not detected, suggesting a complex mechanism. Changes in the transcriptional levels on one type of chromosome were shown previously to result in disruption of transcription on all chromosomes in humans and worms^28,30,66,67^, see discussion). Taken together these results indicate that transcription in the YBT7 germline is broadly misregulated, and many genes from both linear and internal segments are more highly expressed than are the same genes in wild-type gonads.

### Worms with bisected X chromosomes have severe meiotic alterations

**Fig. 4:**
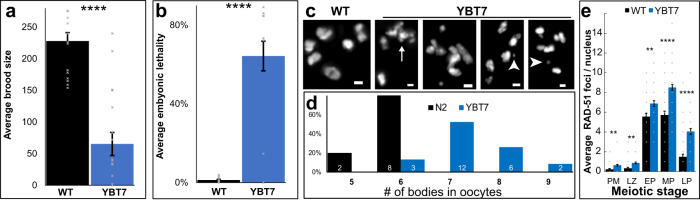
Meiotic defects are present in YBT7. **a** Average ± SEM brood size (*n* = 16 and 18 worms for WT and YBT7) and **b** embryonic lethality (*n* = 16 and 13 worms for WT and YBT7, *p* value = 0. 00006 by the two-tailed Mann–Whitney test) levels for wild-type and YBT7 progeny. **c** Mature wild-type and YBT7 oocytes stained with DAPI. Arrow indicates a chromosome aggregation. Arrowheads indicate chromosomal fragments. **d** Percentages of DAPI-stained bodies (excluding fragments) in wild-type and YBT7 oocytes. The number of scored oocytes is marked on the bars. **e** Average ± SEM RAD-51 foci per nuclei at different oogonial stages. *n* = 40, 41, 47, 40, and 49 nuclei for WT PM pre-meiotic, LZ leptotene/zygotene, EP early pachytene, MP mid-pachytene, and LP (late pachytene, respectively and 60 nuclei for each stage in YBT7. Scale bar = 1 μM. **p < 0.01, ***p < 0.001, ****p < 0.0001, by the two-tailed Mann–Whitney test.

The dramatic transcription misregulation observed in YBT7 gonads suggested that meiosis is disrupted in this strain. Indeed, there was a striking reduction in progeny brood size (Fig. 4a; 230 ± 13 vs. 70 ± 17 per worm for wild type vs. YBT7, respectively, *n* ≥ 16), indicating reduced fertility. Moreover, 64 ± 7% of the embryos laid by YBT7 worms did not hatch (Emb phenotype), whereas only 1.3 ± 0.4 of wild-type embryos did not hatch (Fig. 4b; *n* ≥ 13). This is suggestive of meiotic failure in YBT7 worms that leads to embryonic lethality. We did not detect a high incidence of males (Him phenotype), which has also been associated with failed meiotic segregations^68^.

To obtain insight into the cellular basis of embryonic lethality, we examined DAPI-stained wild-type and YBT7 mature oocytes. Wild-type oocytes almost always contained six DAPI-stained bodies (Fig. 4c), corresponding to the six bivalents of *C. elegans*. Many YBT7 oocytes had chromosomal aggregations, fragments, and univalent-like bodies. The number of DAPI-stained bodies varied from six to nine (excluding chromosomal fragments and aggregations, Fig. 4c, d). These chromosomal aberrations could be due to aberrant repair of DNA double-strand breaks. To test this hypothesis, we stained wild-type and YBT7 gonads with anti-RAD-51 antibodies, which mark homologous recombination repair sites^69–71^. In wild-type gonads, we observed previously described dynamics of RAD-51 foci^69^. The number of foci rose during the leptotene/zygotene stage, reached a maximum during mid-pachytene, and decreased during late pachytene (Fig. 4e). In YBT7 gonads, we observed similar dynamics, but the average values in YBT7 gonads were higher in all stages (Fig. 4e). For example, in mid-pachytene, we found 5.7 ± 0.4 foci per nucleus in wild-type gonads, whereas in YBT7 gonads we found 8.5 ± 0.3 (*n* ≥ 40). Thus, double-strand break repair is perturbed in the YBT7 strain.

It is possible that the lack of silencing of the X segments stems from the increased number of unrepaired double-strand breaks. To test this possibility, we used ionizing radiation to induce DNA double-strand breaks in wild-type gonads. In wild-type irradiated gonads we did not observe increased association of RNAPII on the X chromosome, which was detected in YBT7 mutants (Figs. 4e and S6). Together, these results suggest that the bisection of the X chromosome caused perturbations in double-strand break repair that led to the reduced fertility of the YBT7 worms.

### The meiotic defects in YBT7 do not result from deletions in *deg-1* and *linc-20* loci

To bisect the X chromosome, we had to delete regions encoding of *linc-20* locus and of an intron of *deg-1*. One of these deletions could theoretically cause the meiotic defects we observed in the YBT7 strain. To rule this out, we engineered a gene disruption in *deg-1* (*deg-1*(*huj28*)). This mutation did not lead to reduced brood size or embryonic lethality phenotypes (Fig. S7a, b). Similarly, a strain we engineered with full deletion of *linc-20* had normal brood size and levels of embryonic lethality (Fig. S7c, d) as previously reported^50^. These results suggest that the meiotic defects in YBT7 are not the result of the loss-of-function of either *deg-1* or *linc-20*.

Although we did not detect meiotic defects in strains with mutations in *deg-1* or *linc-20* genes, it is possible that the specific deletions we created in those sites led to the meiotic phenotypes. We, therefore, used homology-directed repair CRISPR engineering^72–80^ to create a strain with the three deletions within the *deg-1* and *linc-20* loci present in YBT7 but without bisection of the X chromosome. The brood size and embryonic lethality of this strain were equivalent to wild type (Fig. S7e, f). Taken together, these results show that the phenotypes of YBT7 are not the result of deletions in *deg-1* and *linc-20* loci.

### The mechanism of reduced X-chromosome silencing in YBT7 is not related to MES-4 but may rely on MES-2

Previous works showed that mutations in several *mes* family genes lead to a reduction in the transient silencing of the X chromosome in hermaphrodite worms^28,30,57^. To test if the reduced silencing observed in YBT7 occurs through a pathway involving the MES proteins, we compared the misregulated genes in YBT7 to the 61 genes reported to be misregulated in *mes-4* mutants^28^. Only two genes are present in both lists (F07D10.1 and F54F7.6). This suggests that the aberrant, transient X chromosome silencing we observed in YBT7 works in parallel to MES-4. To confirm this, we depleted *mes-4* by RNAi-mediated silencing in wild-type and YBT7. We quantified the staining by antibody to RNAPII on the X compared to the autosomes and found a partial additive effect in *mes-4* RNAi YBT7 worms (Fig. S8a, b). In contrast, when we depleted worms of *mes-2*, there was not a significant difference in relative staining on the X compared to autosomes (Fig. S8a, b). Finally, our RNA-seq data do not support the possibility that the effect in YBT7 is a result of a change in expression of *mes* genes as we did not find misregulation of any of the *mes* genes in YBT7 (Supplementary Data 1). In line with these results, the nucleoplasmic localization of MES-2 was unperturbed in the bisected X background (Fig. S8c). Therefore, it is possible that similar to *mes-2*, the reduced silencing observed in YBT7 works via H3K27me3 deposition.

### Aberrant X chromosome transient meiotic silencing occurs in another strain lacking X chromosome continuity

**Fig. 5:**
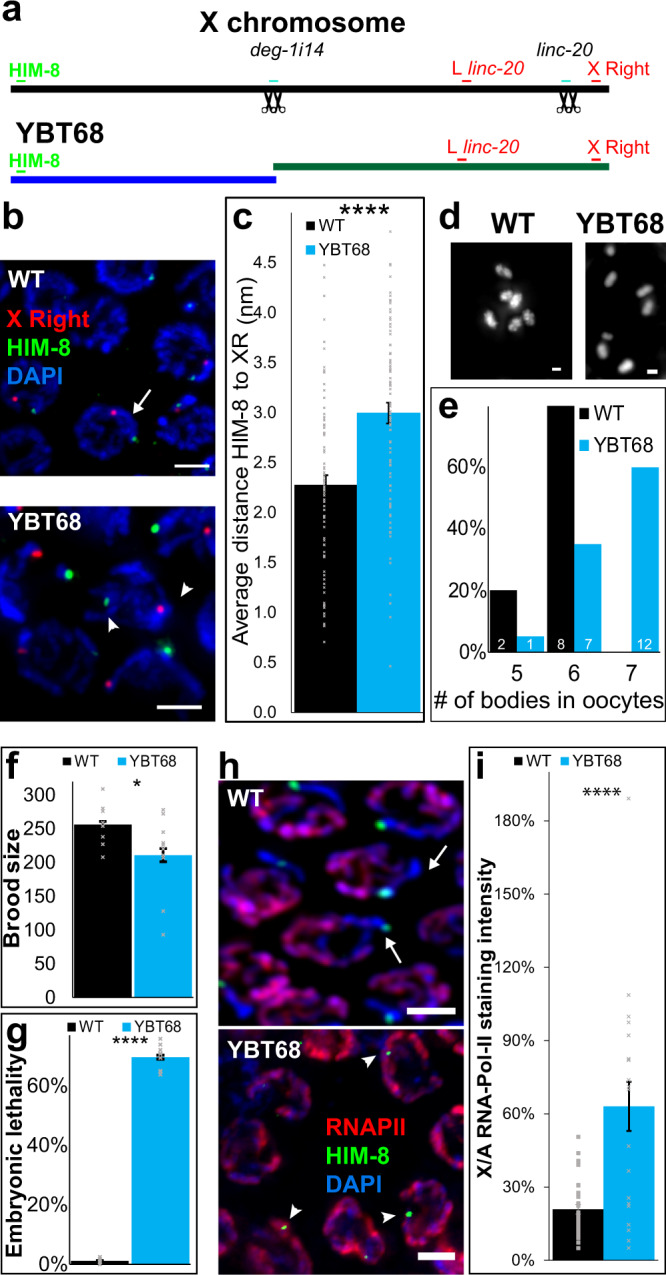
Another engineered loss of X chromosome continuity leads to defects in chromosome silencing and meiosis. **a** Illustration of the X chromosomes in the wild-type and proposed outcome in the YBT68 strain. Sites of gRNAs (black scissors) and cytological markers (green and red) are marked on the schematics. **b** Pachytene nuclei stained with DAPI (blue), anti-HIM-8 (green), and FISH probe directed to the right end of the X chromosome (red). Arrow indicates hybridization of both probes on the same chromosome. Arrowheads indicate probe hybridization on different chromosomes. Scale bar = 3 μM. **c** Averages ± SEM of the distance between HIM-8 and the FISH probe in wild-type and YBT68 pachytene nuclei. *N* = 80 nuclei. **d** Mature wild-type and YBT68 oocytes stained with DAPI. Scale bar = 1 μM. **e** Distribution of the numbers of DAPI-stained bodies in wild-type and YBT68 oocytes. The number of scored oocytes is marked on the bars. **f** Average ± SEM brood size (*p value* = 0.03836 by the one-tailed Mann–Whitney test) and **g** embryonic lethality of wild-type and YBT68 broods. *n* = 9 worms. **h** Early-mid pachytene nuclei from wild-type and YBT68 gonads stained with DAPI (blue), anti-HIM-8 (green), and anti-RNAPII (red). Scale bar = 3 μM. Arrows indicate HIM-8-marked chromosome with no RNAPII staining. Arrowheads indicate HIM-8-marked chromosomes with significant RNAPII staining. **i** Averages ± SEM of relative RNAPII staining levels in wild-type and YBT68 nuclei on HIM-8-marked body vs. an autosome. (*p value* = 0.00094 by the two-tailed Mann–Whitney test, *n* = 31 and 20 nuclei for WT and YBT68, respectively. *p < 0.05 by the one-tailed Mann–Whitney test, ****p < 0.0001, by the two-tailed Mann–Whitney test.

To verify that the phenotypes we observed in YBT7 stem from the bisection of the X chromosome, we engineered another strain, YBT68, in which the X chromosome continuity is lost. We isolated a strain with small deletions within i14 of *deg-1* and within the *linc-20* locus. Nanopore long-read sequencing data suggested that the X chromosome is noncontinuous at the *deg-1* locus, but there was no indication of a chromosomal aberration at the *linc-20* locus or of a translocation. This raised the possibility that the X was bisected at the *deg-1* locus with some overlap between the right and left sides of the break, albeit other options also exist (Fig. 5a and Methods). In line with the bisection hypothesis, signals from anti-HIM-8 antibodies and a FISH probe directed to the right side of the X were present on the same DAPI-stained track in 100% of wild-type pachytene nuclei but only in 4% of YBT68 nuclei (Fig. 5b, *n* = 80, *p* value < 0.00001, Fisher exact test). The HIM-8 and FISH foci were spatially further from each other in YBT68 than in wild-type gonads (Fig. 5b, c, 3.0 ± 0.1 vs. 2.3 ± 0.1 μM, respectively, *n* = 80). Staining with anti-HIM-8 and a probe directed to the left side of *linc-20* locus showed similar results. Further, mature YBT68 oocytes stained with DAPI usually contained seven bodies, whereas the majority of wild-type oocytes had six (Fig. 5d, e). These data indicate that in the YBT68 strain, the X chromosome continuity is lost and it may possibly be bisected at the *deg-1* locus.

Using Illumina next-generation whole-genome sequencing of the parental wild-type strain as well as YBT7 and YBT68 strains at ~100X coverage, we verified that there are no off-target structural alternations or mutations within coding genes that are shared between YBT7 and YBT68 (Table S1). If the transient X chromosome meiotic silencing is dependent on X chromosome continuity, YBT68 and YBT7 should have similar phenotypes. Indeed, the average brood size of YBT68 worms was significantly smaller than that of the wild-type worms (Fig. 5f), and there was over 60% embryonic lethality (Fig. 5g). We note that the progeny numbers for YBT68 were higher than for YBT7, likely because the nature of the chromosomal aberrations in mature oocytes is different (compare Fig. 4 to Fig. 5). This could result in differences in genetic programs that lead to different meiotic outcomes. We verified that the meiotic phenotypes present in YBT68 were not a result of the 13-base pair deletion in i14 of *deg-1* by engineering a strain in which we recreated the wild-type sequence at the i14 locus within the YBT68 strain. This repair did not rescue the brood size or embryonic lethality defects observed in YBT68 (Fig. S7g, h).

Importantly, the loss in the transient X chromosome silencing we observed in YBT7 was also observed in the YBT68 strain: The relative staining for RNAPII on the anti-HIM-8-stained track was significantly higher in YBT68 than in wild-type gonads (Fig. 5h, i). These results show that in both strains we engineered, there were defects in meiotic X chromosome transcriptional silencing.

### Transient X chromosome meiotic silencing is aberrant in a strain with reciprocal translocation of chromosomes V and X

**Fig. 6:**
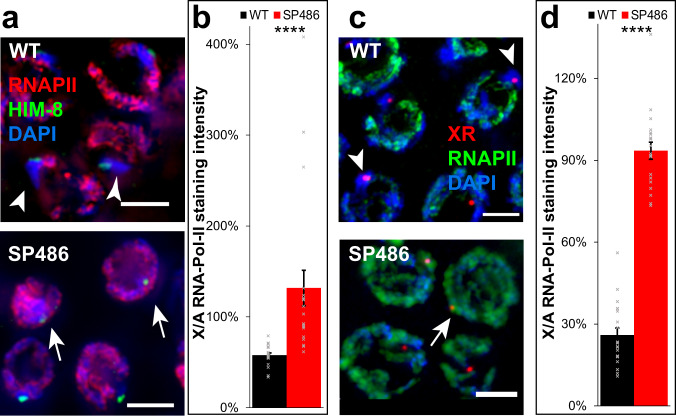
Reciprocal translocation of X and V chromosomes causes defects in X chromosome meiotic silencing. **a** Early-mid pachytene nuclei stained with DAPI (blue), anti-RNAPII (red), and anti-HIM-8 (green). **b** Averages ± SEM of relative RNAPII staining levels in wild-type and YBT68 nuclei on HIM-8-marked body vs. an autosome. **c** Early-mid pachytene nuclei stained with DAPI (blue), anti-RNAPII (green), and FISH probe to the right side of the X (red). **d** Averages ± SEM of relative RNAPII staining levels in wild-type and YBT68 nuclei on X-right marked body vs. an autosome. Scale bar = 3 μM. Arrows indicate chromosomes with no RNAPII staining. Arrowheads indicate chromosomes with significant RNAPII staining. *n* = 20 nuclei. ****p < 0.0001, by the two-tailed Mann–Whitney test.

We next sought to test whether the loss of X chromosome continuity compromised its silencing using a different technology. Herman et al. previously reported the isolation of SP486, a strain with the mnT10 reciprocal translocation between chromosomes X and V^81^. Hermaphrodite worms of this strain have a pair of homologous chromosomes with part of chromosome V fused to part of the X chromosome and another pair with the reciprocally fused parts. We stained gonads of SP486 worms with antibodies to HIM-8 and RNAPII. As in YBT7 and YBT68 early-mid pachytene nuclei, in these nuclei of SP486 the ratio of RNAPII staining between the HIM-8-marked region and the autosomes was significantly higher than in wild-type gonads (Fig. 6a, b, 130 ± 20% vs. 58 ± 3%, respectively).

To the best of our knowledge, the break sites that led to the translocation in the SP486 strain have not been fully characterized. It is therefore possible that the region that binds to HIM-8 is only a small part of the X chromosome and most of the X was translocated to a different part of chromosome V and was silenced. To test this possibility, we stained gonads with antibodies against RNAPII and with FISH probes directed to the right side of the X chromosome. In wild-type early-mid pachytene nuclei, staining for RNAPII was very weak on the right side of the X chromosome compared to that in autosomes (Fig. 6c, d). In SP486 gonads, however, the region stained by the probe directed to the right side of the X chromosome was stained for RNAPII as strongly as were autosomes (Fig. 6c, d, 26 ± 3% vs. 94 ± 3% for wild type and SP486, respectively, *n* = 20). These results indicate that silencing initiation fails when regions of the X chromosome are translocated through natural events. Moreover, these results indicate that silencing aberrations due to continuity loss are not limited to specific break sites.

### Both synapsed and unsynapsed segments of the X chromosome are actively transcribed in early meiotic stages

The accepted model for MSCI places the trigger for the inactivation in the unsynapsed region of the sex chromosomes^17^. In hermaphroditic *C. elegans*, the two X chromosomes synapse, yet undergo transcriptional silencing during early oogenesis^26–29,56^. MSUC also occurs in hermaphroditic worms^33^. If MSUC occurs on the bisected X chromosome, we expected that unsynapsed segments of the X would be silenced. Alternatively, if unsynapsed segments do not undergo silencing upon bisection of the X chromosome, the mechanism may differ from MSUC. Due to the nature of pairing in *C. elegans* meiosis, which is required for homolog synapsis, any chromosomal body that harbors a pairing center, pairs and synapses, whereas bodies without paring centers often do not synapse^53,82^. The linear segment of YBT7 contains the pairing center and is therefore expected to pair and synapse, whereas the internal segment is expected to stay unsynapsed or undergo self-synapsis. Our data indicate that genes on both the linear and internal segments of the X chromosome are highly upregulated in YBT7 (Fig. 2g). Therefore, either the internal segment is synapsed or it escapes MSUC.

**Fig. 7:**
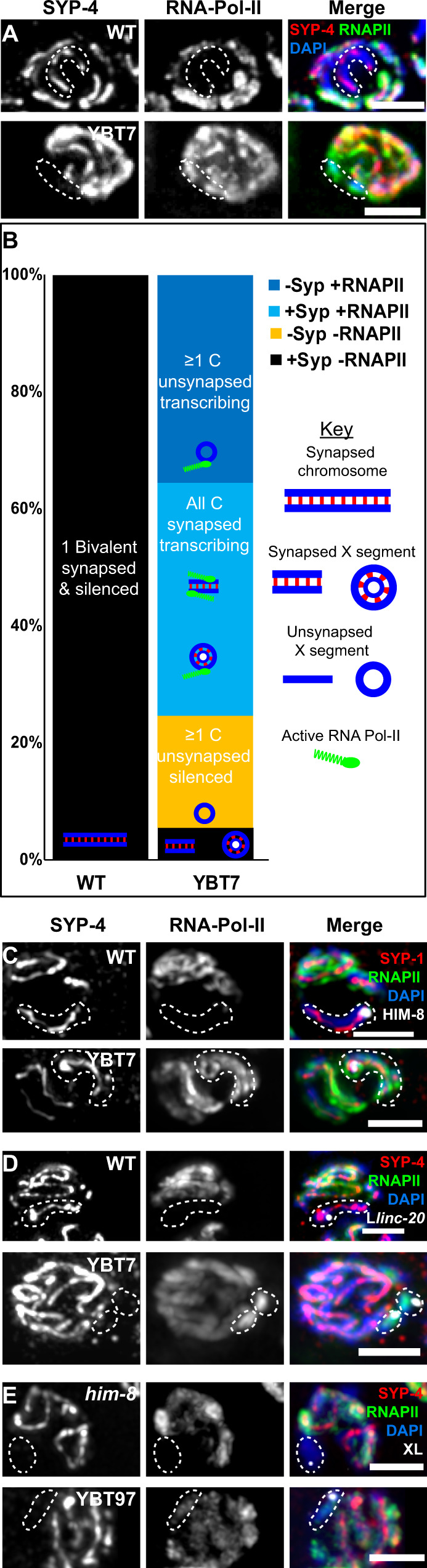
Unsynapsed X segments are transcriptionally active. **A** Mid-pachytene nuclei of wild-type and YBT7 genotypes stained with DAPI (blue), anti-RNAPII (green), and anti-SYP-4 (red). Scale bar = 3 μM. *n* = 42 and 73 nuclei for WT and YBT7, respectively. **B** Distribution of the percentages of wild-type and YBT7 early and mid-pachytene nuclei with a chromosome stained for SYP-4 but not RNAPII (black), one or more chromosomes lacking SYP-4 and RNAPII staining (yellow), all chromosomes stained with SYP-4 and RNAPII (light blue), and one or more chromosome stained without SYP-4 and with RNAPII (blue). Possible interpretations for the different categories are illustrated on the bars: In wild type, the synapsed chromosome that does not stain with RNAPII is probably chromosome X, and in YBT7 these are the synapsed X segments (black). In YBT7, the chromosomes without SYP-4 and RNAPII are segments of the X that are not synapsed and are silent (yellow). Nuclei that only contain chromosomes with SYP-4 and RNAPII have either lost the internal segment or both segments are synapsed and active (light blue). The chromosomes that stain with RNAPII but not with SYP-4 are the internal segments that cannot synapse and that escape both sex chromosome silencing and MSUC (blue). **C** Mid-pachytene nuclei stained with DAPI (blue), anti-RNAPII (green), anti-SYP-1 (red), and anti-HIM-8 (white). *n* = 30 nuclei. **D** Mid-pachytene nuclei stained with DAPI (blue), anti-RNAPII (green), anti-SYP-4 (red), and left *linc-20* (white). *n* = 27 and 40 nuclei for WT and YBT7, respectively. **E** Mid-pachytene nuclei stained with DAPI (blue), anti-RNAPII (green), anti-SYP-4 (red), and X left FISH probe (white). Dashed lines: HIM-8 or FISH marked body *n* = 21 and 47 nuclei for *him-8* and YBT97, respectively. Scale bar = 3 μM.

To determine if unsynapsed segments are silenced or not, we stained gonads with antibodies directed against the synaptonemal complex central protein SYP-4^83^ and against active RNAPII. In 100% (*n* = 42) of wild-type early and mid-pachytene nuclei, we found a DAPI-stained body with an SYP-4 track but without significant RNAPII staining, corresponding to the synapsed and silenced X chromosomes present in wild-type gonads (Fig. 7A, B). In YBT7 gonads, only 5% (*n* = 4/73) of mid-pachytene nuclei had this type of staining combination (*p* < 0.0001, Fisher’s exact test). In 40% (*n* = 29/73) of YBT7 early and mid-pachytene nuclei, all the chromosomes were stained with both anti-SYP-4 and anti-RNAPII. These nuclei have either lost the internal segment, which does not contain a pairing center, or the internal segment was synapsed (Fig. 7C and see discussion). In 36% (*n* = 26/73) of the YBT7 nuclei, we detected chromosomes stained with anti-RNAPII but not anti-SYP-4 (Fig. 7A, B). These chromosomes were mostly smaller than other chromosomes (Fig. 7A), suggesting that they are segments of the X chromosome. Quadruple staining of YBT7 gonads with DAPI, anti-RNAPII, anti-SYP-1, and anti-HIM-8 indicated that chromosomal bodies stained with anti-RNAPII but not with the antibody to central element protein SYP-1 were never stained with anti-HIM-8 (*n* = 30, Fig. 7C). This shows that these unsynapsed active segments are not the linear segment of the X. When we used the FISH probe directed to the left of the *linc-20* locus, we found that the internal segment was marked with SYP-4 in some cases. More importantly, we detected chromosomal bodies marked by this FISH probe and stained with anti-RNAPII but not with anti-SYP-4 (Fig. 7D). These results suggest that the internal segment can in some cases be transcribed even when not synapsed.

Our cytological and Nanopore results suggest that the internal segment exists as a circular element (Figs. 1e and S1). It is, therefore, possible that transcription of unsynapsed chromatin is a unique feature of this special chromosomal topology and not an outcome of the bisection. To determine whether this was the case, we prevented the linear segment from undergoing synapsis in the YBT7 background by introducing a mutation within *him-8*. We stained the gonads of strains with *him-8* mutations in the YBT7 and wild-type backgrounds for SYP-4 and RNAPII. In the wild-type background, despite mutation in *him-8*, unsynapsed X chromosomes in mid-pachytene were never associated with staining for RNAPII. In contrast, we did observe unsynapsed linear segments with significant RNAPII staining in mid-pachytene nuclei when the *him-8* mutation was present in the YBT7 background (Fig. 7E). Thus, linear X chromosome segments can escape MSUC.

The ability to escape MSUC may not be a unique feature of the X chromosome. To gain insight into whether loss of autosome continuity can also lead to reduced MSUC, we tested worms heterozygous for the nT1 IV:V reciprocal translocation^84,85^. In pachytene nuclei of these worms, unsynapsed chromatin was never associated with RNAPII (Fig. S9). Together, these results indicate that segments of the X chromosome in YBT7 were not silenced regardless of whether or not they were synapsed. Our findings support the hypothesis that loss of sex chromosome continuity can prevent both X chromosome transient meiotic silencing and silencing of unsynapsed chromatin. Whether these mechanisms are linked or mechanistically independent remains unclear.

## Discussion

Sex chromosomes have emerged several times during animal evolution^86,87^ as has meiotic silencing^13–17,88^. This suggests that evolutionary pressure drives the silencing of sex chromosomes during meiosis. Since MSUC occurs in organisms without sex chromosomes, it is conceivable that when sex chromosomes emerge, they undergo silencing in heterogametic meiocytes simply because they do not synapse as was suggested previously^17^. Supporting this model are observations in various organisms that there is aberrant MSCI and synapsis in sex chromosomes that have undergone translocation with an autosome^35–38^.

In this work we created a *C. elegans* strain in which the X chromosome was bisected into segments of similar size to test whether superimposed on the synapsis trigger for meiotic silencing, there is another mechanism that depends on sex chromosome continuity. Cytological and quantitative transcriptomic evaluation of the YBT7 strain, in which the X chromosome is bisected, support our conclusion that there are defects in meiotic silencing in the two X-chromosome segments. Although X-linked genes were highly enriched among the differentially expressed genes in the YBT7 strain, a considerable number of autosomal genes were also upregulated. Differential expression of autosomal genes could be due to misregulation of genes on the X chromosome that directly influence autosomal transcription (e.g., transcription factors and chromatin modifiers). Although genes that fall into this category were upregulated (e.g., *lsd-1* and *atf-5*) in the YBT7 strain, we were not able to link these directly to upregulated genes on the autosomes. Previous studies of worms and humans showed that changes in transcription on one chromosome can lead to opposite effects on the affected chromosome and differential expression of genes on other chromosomes^28,30,66,67^. For example, in trisomy 21 cells, the vast majority of the differentially expressed genes are on other chromosomes^66,67^. A meta-analysis showed that among all upregulated genes in trisomy 21 cells, only about 7% of genes are transcribed from chromosome 21^89^. Similar to YBT7, in trisomy 21 cells a low percentage of genes on the impacted chromosome are downregulated. Interestingly, as is the case for YBT7, most of the genes downregulated on chromosome 21 in trisomy 21 cells are mapped to the q arm and only one is located on the p arm^89^. Together these data suggest that the change in the transcription levels of X-linked genes in YBT7 led to global alterations in the transcriptome through a complex and as-yet-unidentified network.

An alternative model for the reduced silencing of the X segments in YBT7 could be a change in the copy number of the segments. As the internal segment may be circular, its segregation and/or replication may be interrupted. We believe this is unlikely to be the mechanism underlying this phenomenon for the following reasons: First, both our Illumina and Nanopore DNA sequencing data are consistent with no change in copy number. Second, it is unclear how a change in the content of a segment that is transcriptionally silenced could lead to the cancelation of this silencing. Third, quantification of the foci stained with the FISH probe directed to the left of the *linc-20* locus in pachytene indicated that 97% of the nuclei contain one or two foci and only 3% of the nuclei contained three foci (Fig. S4). Fourth, YBT68 and SP486 do not have a circular element but characteristics are virtually identical to those of YBT7. Thus, our hypothesis is that the bisection of the X chromosome resulted in the reduced silencing observed in YBT7.

We also provide evidence that a reciprocal translocation of the X with an autosome present in SP486 worms leads to defects in the transient silencing of the X. Kelly et al. reported that in pachytene nuclei of this strain, a region of a chromosome had low levels of H3K4me2^26^. We quantified the levels of RNAPII in autosomes and in the two segments of the X chromosome in early-mid pachytene nuclei and found that levels of transcription were significantly increased in the X chromosome regions. This discrepancy could be due to several factors. It is possible that the X-related segments in SP486 gonads are not enriched with histone modifications correlated with transcription, even though transcription is occurring. Alternatively, since Kelly et al. did not use cytological markers to identify chromosomes, it is possible that the segments they observed were not part of the X, but rather an unsynapsed part of chromosome V. We noticed high variability of anti-RNAPII staining on the X segments in the SP486 nuclei, and only reached our conclusion following careful quantification. This type of variability in meiotic silencing markers of translocated X segments is not limited to *C. elegans*. For example, Turner et al. used γH2AX as a marker for MSCI initiation in mouse testes with a reciprocal translocation of chromosomes 16 and X and found that in only about half of pachytene spermatocyte nuclei with synapsed X^16^ was the X part stained for γH2AX^38^. Thus, in the system studied by Turner et al., the X silencing occurs in about 50% of synapsed X chromosome regions. Similarly, Mary et al. reported that in a boar with translocation of chromosomes 13 and Y, about 50% of the Y and X chromosomes showed no γH2AX signal in pachytene spermatocytes nuclei^39^. Similar levels were reported by Barasc et al. in a boar with translocation of chromosomes 1 and Y^36^. These reports indicate that in both worms and mammals, reciprocal translocation of a sex chromosome with an autosome incompletely perturbs meiotic silencing (MSUC, MSCI, or the transient silencing present in hermaphrodite worms). The variability in the silencing observed in translocations involving sex chromosomes and autosomes could also arise due to the dynamics of epigenetic modifications.

Our finding that in some cases the unsynapsed segments of the X chromosomes were not silenced was surprising given the prevalence of MSUC in many organisms. This uncoupling of synapsis and silencing of chromatin derived from sex chromosomes may be due to an epigenetic mechanism. It will be important to determine whether this uncoupling of synapsis and expression is unique to *C. elegans* hermaphrodites in which the X chromosomes do synapse yet still undergo silencing. Several lines of evidence suggest that this feature is evolutionarily conserved. First, silencing of unsynapsed chromatin has been observed in *C. elegans*^33^, so the basic mechanism of MSUC exists. Second, bisection of sex chromosomes in mammals leads to silencing of synapsed parts of autosomes and sex chromosomes in pachytene^35–37^. Third, in early pachytene nuclei of XO mice, the unsynapsed X chromosome is marked by γH2AX yet is transcribed^31^. Taken together these reports imply that sex chromosomes undergo silencing in heterogametic wild-type meiocytes not solely due to their unsynapsed state.

Considering these previous reports and the findings we report here, we propose the following model: Under normal conditions, meiotic silencing of the sex chromosomes is activated on complete sex chromosomes in heterogametic meiocytes, as well as in the early stages of hermaphrodite worms. Lack of synapsis in autosomes leads to silencing, whereas aberrant synapsis of sex chromosomes cancels their silencing in heterogametic cells. When sex chromosomes are segmented, at least in *C. elegans*, another mechanism is activated, and the silencing of sex chromosomes is perturbed. This mechanism can in some meiocytes override MSUC, and the unsynapsed segments are transcribed.

What evolutionary drive links silencing of sex chromosomes to their continuity? One possible answer comes from inherent problems with the DNA repair of heterogametic chromosomes during meiosis. As interhomolog recombination is the preferred repair pathway of meiotic breaks, heterogametic chromosomes are at risk of aberrant repair and breakage. Therefore, loss of MSCI when sex chromosome continuity is compromised may simply be a safety mechanism that eliminates meiocytes in which the sex chromosomes are segmented. The continuity loss will in turn disrupt MSCI, which will lead to gametogenesis failure and apoptosis. Alternatively, flexibility in the dichotomic chromosome state between silent sex chromosomes and active autosomal chromosomes may allow the formation and disappearance of sex chromosomes with evolutionary progression.

Like initiation of silencing, other meiotic processes are also executed differently on the X chromosome than on autosomes in hermaphroditic worms^90–95^. These differences suggest that the X chromosome is marked differently than the autosomes, and, indeed, previous reports indicate that the X chromosomes are enriched with different histone modifications than are autosomes^26,33,90,91,96^. Thus, an epigenetic mechanism may regulate silencing and its dependence on continuity. Our results suggest the possibility that the silencing initiation depends on an output of sex chromosome that assess their continuity. One potential regulator is the synaptonemal complex. Axis length in *C. elegans* appears to regulate DNA double-strand break formation and crossover interference^97,98^, and axis proteins are in close contact with chromatin. A recent report also showed that axis proteins control the different dynamics of meiotic recombination in small chromosomes in yeast^99^. Additional studies should test these possibilities to determine if these regulators connect X chromosome continuity to meiotic silencing.

## Methods

### Strains and alleles

All strains were cultured under standard conditions at 20 °C unless specified otherwise^100^. The N2 Bristol strain was utilized as the wild-type background. Worms were grown on NGM plates with *Escherichia coli* OP50^100^. All experiments were conducted using adult hermaphrodites 20–24 h after the L4 stage. The following mutations and chromosome rearrangements were used: *mes-2*(*ax2059*[*mes-2::GFP*]), *him-8*(*e1489*), nT1 (IV:V), and SP486: mnT10 (V;X)^81^. Strains engineered in this work (see below): YBT7: *deg-1*(*huj32*) *linc-20*(*huj2*) *hujCf1*, YBT68: *deg-1*(*huj33*) *linc-20*(*huj29*) *hujCf2*, YBT54: *linc-20*(*huj21*), YBT67: *deg-1*(*huj28*), YBT75: *linc-20*(*huj29*), YBT72: *deg-1*(*huj32*) *linc-20*(*huj2*), YBT97: *him-8*(*e1489*); *deg-1*(*huj32*) *linc-20*(*huj2*) *hujCf1*, YBT98*: mes-2*(*ax2059*[*mes-2::GFP*]); *deg-1*(*huj32*) *linc-20*(*huj2*) *hujCf1*.

### Generation of strains by CRISPR-Cas9 genome engineering

To generate the YBT7 strain, we used the procedure described in^52^ with the modifications detailed previously^51^. The gRNA sequences are given in Table S2. Worms were isolated based on PCR analysis of targeted loci, and segmented chromosomes were identified via Nanopore sequencing. The YBT7 strain carries the deletions *deg-1*(*huj32*) X:7769748-7772344 and *linc-20*(*huj2*) X:16508000-16509415 and X:16511093-16513798. A fusion of the region X:~7772k to X:~16511k and fusion of the segment left of X:7774k to segment right of 16507k were confirmed by Sanger sequencing. Illumina DNA sequencing indicated that there are other structural aberrations and mutations, but these were not confirmed by Sanger sequencing.

All other strains were generated using the protocol described previously^79^ with the modifications detailed previously^51^. YBT68: *deg-1*(*huj33*) *linc-20*(*huj29*) *hujCf2* was generated using CRISPR RNAs (crRNAs) listed in Table S2, isolated in a similar strategy to YBT7, and outcrossed five times. This strain carries the deletions *deg-1*(*huj33*) X:7769768–X:7769760, *linc-20*(*huj29*) X:16507326–16508724 and X:16509401–16510374. Seventeen reads of the Nanopore long-reads sequencing data (>2000 bases) of YBT68 cover the region of X:7,759,000–7,760,000 (upstream to deg-1). Of these, seven reads ended on the left side directly at X:7,759,562 ± 8 (vs. 0/20 in the wild-type strain). Within *deg-1*, 21 long reads covered the region of X:7,773,000–7,774,000 in YBT68. Seven of those long reads ended on the right-side at X:7,773,787 ± 5 (vs. 0/26 in wild type). In wild-type, there were single reads that covered the entire region of X:7,759,500 and X:7,774,000, whereas in YBT68 no reads spanned this region even though the coverage at this locus was >X15, and many of the reads in this region were more than 10,000 bases long. No read had significant alignment to any other genomic locus. These data suggested that the right and the left sides of the X are not connected, and cytological observation supported this hypothesis. Illumina DNA sequencing suggested the presence of other structural aberrations and mutations, but these were not confirmed by Sanger sequencing.

YBT54: *linc-2*(*huj21*) was generated using crRNAs listed in Table S2 and was outcrossed five times. This strain carries the deletion *linc-20*(*huj21*) X:16507934–16509755. YBT67: *deg-1*(*huj28*) was engineered using crRNAs listed in Table S2. It has a four-base out-of-frame deletion at position 297 of the first exon. YBT75: *linc-20*(*huj29*) was engineered using appropriate crRNAs and single-stranded oligodeoxynucleotides (ssODNs) (Table S2). These were injected into YBT68 worms, and repair of the *deg-1* genotype was verified by sequencing.

YBT72 was engineered through three CRISPR engineering steps as follows: Wild-type worms were engineered using crRNAs “homologous lincs 5ʹ crRNA” and “homologous lincs 3ʹ crRNAs” together with the ssODN “Linc-20 del1-ssODN” and “Linc-20 del2-ssODN”. The strain with *linc-20* (*huj21*) was identified by PCR and verified by sequencing. After one outcross with the wild-type strain, worms were further engineered with “Linc-20 YBT7 del-2 5′ crRNA” and “Linc-20 YBT7 del-2 3′ crRNA” together with “Linc-20 del2-ssODN”, and worms with *linc-20*(*huj2*) were identified by PCR and verified by sequencing and outcrossed once with the wild-type strain. A strain with *deg-1*(*huj32*) was engineered by injecting wild-type worms with crRNAs “Deg-1 YBT7 del- 5′ crRNA” and “Deg-1 YBT7 del- 3′ crRNA” and ssODN “Deg-1 del-ssODN” (Table S2) and outcrossed once. Worms with *deg-1*(*huj32*) were crossed with worms with *huj*(*2*) to establish YBT72. All the engineered mutations were verified by Sanger sequencing.

### Cytological analysis and immunostaining

DAPI staining and immunostaining of dissected gonads were carried out as described^69,101^. Worms were permeabilized on Superfrost+ slides for 2 min with methanol at −20 °C and fixed for 30 min in 4% paraformaldehyde in phosphate‐buffered saline (PBS). Staining with 500 ng/ml DAPI was carried out for 10 min, followed by destaining in PBS containing 0.1% Tween 20 (PBST). Slides were mounted with Vectashield anti-fading medium (Vector Laboratories). Primary antibodies were used at the following dilutions: rabbit anti-SYP-4 (1:200, kind gifts from S. Smolikove, The University of Iowa), goat anti-SYP-1 (1:200, kind gifts from S. Smolikove, The University of Iowa), rabbit anti-HIM-8 (Novus Biological cat # 41980002, 1:2000), rat anti-HIM-8 (1:100, a kind gift from A. Dernburg, University of California, Berkeley), rabbit anti-H3K27me3 (Millipore cat # 07-449, 1:1000), rabbit anti-H3K4me3 (Millipore cat # 05-745, 1:1000), mouse anti-H3K9me2 (Abcam cat # ab1220, 1/200), rabbit anti-H4K20me (Abcam cat # ab9051, 1/200), rabbit anti-H3K36me3 (Abcam cat # ab9050, 1/200), and mouse anti-pSer2 RNAPII (Diagenode cat # C15200005, 1:1000). All secondary antibodies used were purchased from Jackson ImmunoResearch Laboratories, and used at 1/200 dilution: Cy2-donkey anti-rabbit (AB_2340612), Cy3-goat anti-rabbit (AB_2338000), Cy3-donkey anti-goat (AB_2307351), Cy2-goat anti-rat (AB_2338278), Cy2-goat anti-mouse (AB_2338746), Cy3-goat anti-mouse (AB_2338690), Cy5-donkey anti-mouse (AB_2338746), Cy5-donkey anti-rabbit (AB_2340607).

### DNA FISH

Probes were made from cosmids provided by the *C. elegans* sequencing consortium at the Sanger Centre. Cosmid DNAs that harbor 30–40 kb of sequence around the chosen genomic target were labeled after linearization by nick translation using Cy3-dUTP (GE Healthcare) as described^102^. For the region left of *linc-20* we used C09G1, for the left side of the X we used F13C5, and for the right side of the X chromosome we used T27B1.

Worms were transferred to a 15-μL drop of egg buffer (118 mM NaCl, 48 mM KCl, 2 mM CaCl_2_, 2 mM MgCl_2_, 5 mM HEPES [pH 7.4])^103^, containing 15 mM NaN_3_ and 0.1% Tween-20 on a 22 mm × 22 mm coverslip. Gonads were dissected and fixed in 3.7% formaldehyde in PBST. A SuperFrost Plus slide (ThermoFisher Scientific) was placed on the coverslip, then frozen on an aluminum block immersed in liquid nitrogen. The coverslip was cracked off and slides were transferred to methanol at −20 °C for 30 min. Slides were washed in 2X SSCT (3 M NaCl, 0.3 M sodium citrate, pH 7, 0.1% Tween20), 25% formamide/2X SSCT and incubated for 4 h at 37 °C in 50% formamide/2X SSCT in a humid chamber. Slides were prehybridized on a heat block at 93 °C for 90 s in hybridization solution (50% formamide, 3× SSC, 10% dextran sulfate) containing 1 μl of the labeled probe. Slides were hybridized overnight in a humid chamber at 37 °C. After washing with 2X SSCT, the slides were either directly labeled with DAPI and mounted in Vectashield solution for visualization or were blocked for 30 min at room temperature in 1% BSA before antibody labeling.

Copy number analysis of chromosomal segments was conducted on stained gonads. Due to partial penetration inherent to this assay, only nuclei with at least one FISH signal were counted.

### Imaging and microscopy

Z-stack 3D images shown in Fig. 2a, b were acquired at 0.3-µm increments using an Olympus FV1000 Inverted Confocal IX81 Microscope and FV10-ASW 3.1 Software (Olympus). All other images were acquired using the Olympus IX83 fluorescence microscope system. Optical Z-sections were collected at 0.30- or 0.60-µm increments with the Hamamatsu Orca Flash 4.0 v3 and CellSens Dimension imaging software (Olympus). Pictures were deconvolved using AutoQuant X3 (Media Cybernetics).

### Progeny and embryonic lethality quantification

Brood sizes and embryonic lethality were determined by placing individual L4 worms on seeded NGM plates, transferring each worm to a new plate every 24 h, and counting embryos and hatched progeny during a 3-day period.

### Analysis of synapsis and expression interactions

Early and mid-pachytene nuclei stained with DAPI, anti-SYP-4, and anti-pSer2 RNAPII were captured at 0.3 μM optic Z intervals. Nuclei were binned into one of four categories: (1) at least one chromosome-positive for RNAPII and negative for SYP-4, (2) all chromosomes positive for both markers, (3) all SYP-4-negative chromosomes also negative for RNAPII, and (4) all chromosomes SYP-4 positive and at least one RNAPII negative. For quadruple staining, only nuclei stained with all four markers were analyzed. X-marked chromosomal bodies were classified as either positive or negative for SYP-4 staining and then the level of RNAPII was evaluated.

### RNA-seq

Gonads were manually dissected from worms at 24 h post L4 and immediately placed in Eppendorf tubes with Trizol reagent. After several freeze-crack cycles in liquid nitrogen, total RNA was extracted using the Zymo Research Direct-zol RNA Miniprep Plus kit. Synthesis of first-strand was done from 10 μg of total RNA using ThermoFisher SuperScript III Reverse Transcriptase with the following primer that includes the T7 promotor, a unique molecular identifier, UMI and polyT: 5′-CGATGACGTAATACGACTCACTATAGGGATACCACCATGGCTCTTTCCCTACACGACGCTCTTCCGATCTNNNNNNNNNNTTTTTTTTTTTTTTTTTTTVN-3′. Removal of excess primers was done using New England Biolabs Exonuclease I and ThermoFisher FastDigest HinfI in provided buffers; samples were incubated 45 min at 37 °C and then 10 min at 80 °C. The product was purified using Beckman AMPure XP magnetic beads, eluted in 14.5 µL of 10 mM Tris, followed by second-strand cDNA synthesis using New England Biolabs NEBNext Ultra II Non-Directional RNA Second Strand Synthesis Module. Samples were concentrated to 8 µL, and then the product was transcribed with the New England Biolabs HiScribe T7 High Yield RNA Synthesis Kit. RNA was purified using AMPure XP beads and eluted in 20 µL of 10 mM Tris. A 9-µL aliquot of RNA was fragmented using Invitrogen RNA Fragmentation Reagents kit for 3 min. Fragments were purified using AMPure XP beads and eluted in 11 μL 10 mM Tris. Synthesis of the first-strand cDNA and was performed using ThermoFisher SuperScript III Reverse Transcriptase using PvG748 primer 5′-AGACGTGTGCTCTTCCGATCTNNNNNN-3′. After purification using AMPure XP beads and elution with 12.5 µL 10 mM Tris, libraries were amplified using Kapa Biosystems HiFi HotStart ReadyMix, with 2p fixed primers (2p Fixed, 5′-AATGATACGGCGACCACCGAGATCTACACTCTTTCCCTACACGACGCTCTTCCGATCT-3′ and 2p Fixed +barcode, 5′-CAAGCAGAAGACGGCATACGAGATNNNNNNNNGTGACTGGAGTTCAGACGTGTGCTCTTCCGATCT-3′). The product was purified using AMPure XP beads and eluted in 32 µL of double-distilled water. Deep sequencing was carried out on an Illumina NextSeq following the manufacturer’s protocols; >38 million reads were generated for each sample.

### Differential expression analysis

Raw reads were trimmed off low quality and technical bases. Cutadapt, version 1.12, with parameters -O 1, -m 15, and --use-reads-wildcards. Reads with overall low quality were removed using fastq_quality_filter, FASTX version 0.0.14, with parameters -q 20 and -p 90. Processed reads were aligned to the *C. elegans* genome version WBcel235 using TopHat2, version 2.1.1. Alignment allowed two mismatches and five base gaps and used gene annotations from Ensembl release 36. Raw counts per gene were calculated with htseq-count, version 0.6.0, using default parameters.

Normalization and differential expression were calculated with the R package DESeq2, version 1.12.4. Calculations were done for genes with at least three raw counts using default parameters. Genes were taken as differentially expressed if their baseMean was above 5 and if the absolute maximum likelihood estimate of the fold change (without shrinkage, lfcMLE) was greater than 5/baseMean^0.5 + 1. This baseMean-dependent threshold for the change in expression required at least twofold change in expression for highly expressed genes, and the requirement becomes stricter as the level expression becomes lower. The MA plot is illustrated in Fig. S5.

### Nanopore DNA sequencing

Worms were washed from NGM plates with M9 buffer, and young adult worms were isolated on a 60% sucrose bed. Worms were then washed in M9 buffer and frozen in liquid nitrogen. DNA was isolated using the Zymo Research Quick-DNA Miniprep kit.

Genomic DNA was barcoded without fragmentation using Oxford Nanopore Technologies EXP-NBD103, SQK-LSK108/9 according to the vendor’s instructions. Approximately 260 ng DNA of each strain was loaded into MinION flowcells (Oxford Nanopore Technologies), and sequencing was performed using the GridION device and MinKnow software for 48 h.

Reads were quality filtered using NanoFilt (version 2.6.0, parameters ‘-q 5 -l 100 –headcrop 40’). Filtered reads were aligned to the *C. elegans* genome (WBcel235) using minimap2, version 2.17^104^. A combination of three tools were used for the identification of structural variations: sniffles, version 1.0.11^105^, NanoSV, version 1.2.3^106^, and SVIM, version 1.2.0^107^. Copy number variations were identified using the R package QDNAseq^108^.

### Illumina DNA sequencing

DNA was extracted from 25 µL of packed young adult worms using Gentra Puregene Tissue Kit (Qiagen) according to the vendor protocol for *C. elegans*. For each sample, 1000 ng of DNA was sheared using the Covaris E220X sonicator. End repair was performed in an 80-µL reaction at 20 °C for 30 min. After purification using AMPURE XP beads in a ratio of 0.75X beads to DNA volume, A bases were added to both 3′ ends followed by adapter ligation in a final concentration of 0.125 μM. A solid-phase reversible immobilization (SPRI) bead cleanup in a ratio of 0.75X beads to DNA volume was performed, followed by eight PCR cycles using 2X KAPA HiFi ready mix in a total volume of 25 µL with the following program: 2 min at 98 °C, eight cycles of 20 s at 98 °C, 30 s at 55 °C, 60 s at 72 °C, and 10 min at 72 °C.

Libraries were evaluated by Qubit and TapeStation. Sequencing libraries were constructed with barcodes to allow multiplexing of four samples on one lane. Between 38–45 million paired-end, 150-bp reads were sequenced on Illumina Nextseq 500 instrument Mid output 300 cycles kit.

Reads were mapped to the *C. elegans* genome (Ensembl’s WBcel235) using the bwa-0.7.5a^109^ mem algorithm and then deduplicated using Picard tools, version 2.8.1. Variant calling was done with GATK’s Haplotype caller, version 3.7^110^. Variants were filtered with the following values for single-nucleotide polymorphisms: QD < 2.0, FS > 60.0, MQ < 40.0, HaplotypeScore > 13.0, MQRankSum < −12.5, and ReadPosRankSum < −8.0 and QD < 2.0, FS > 200.0, and ReadPosRankSum < −20.0. Variants were then annotated with Ensembl’s Variant Effect Predictor, version 83^111^.

### Measuring distances between chromosome markers

To measure the spatial distance between chromosomal makers, mid-pachytene nuclei positively stained with DAPI, anti-HIM-8, and FISH probe were completely captured at 0.3-µm Z increments. The distance between the HIM-8 foci and the FISH probe was measured using ImageJ. When two foci of a specific probe were detected in the same nucleus, we scored the shortest distance between the different probes. Significance was estimated via the Mann–Whitney test.

### Relative staining intensity of expression markers of X chromosome vs. autosomes

To measure the level of expression marker staining, early pachytene nuclei were positively stained for DAPI and with antibodies to H3K27me3, H3K4me3, H3K9me2, H3K20me, H3K36, or RNAPII were analyzed. The staining level on the X chromosome (marked by either HIM-8 or the FISH probe directed to the right side of the X) was measured in ImageJ, as well as on another chromosome within the same nucleus. The ratio for each nucleus was calculated and averaged across all nuclei.

### RNAi

Feeding RNAi experiments were performed at 20 °C as described previously^112,113^. Control worms were fed HT115 bacteria carrying the empty pL4440 vector. A feeding vector from the *C. elegans* RNAi collection (Source Biosciences) was used to deplete *mes-4* and one from the ORFeome RNAi collection^114^ was used to deplete *mes-2*.

### Reporting Summary

Further information on research design is available in the Nature Research Reporting Summary linked to this article.

## Supplementary information

Supplementary figures and tables

Supplementary Data 1

Description of additional supplementary files

Reporting Summary

## Data Availability

Table S2 contains detailed descriptions of all primers used for genome engineering and genotyping. Source RNASeq data for Fig. 2g are provided with this paper at NCBI’s Gene Expression Omnibus, under accession number GSE171938. Source data are provided with this paper.

## Source data

source data file

## References

[1] Couteau F, Goodyer W, Zetka M (2004). Finding and keeping your partner during meiosis. Cell Cycle.

[2] Gerton JL, Hawley RS (2005). Homologous chromosome interactions in meiosis: diversity amidst conservation. Nat. Rev. Genet.

[3] Gray S, Cohen PE (2016). Control of meiotic crossovers: from double-strand break formation to designation. Annu Rev. Genet..

[4] Jasin M, Rothstein R (2013). Repair of strand breaks by homologous recombination. Cold Spring Harb. Perspect. Biol..

[5] Mezard C, Jahns MT, Grelon M (2015). Where to cross? New insights into the location of meiotic crossovers. Trends Genet..

[6] Page SL, Hawley RS (2004). The genetics and molecular biology of the synaptonemal complex. Annu Rev. Cell Dev. Biol..

[7] Rog O, Dernburg AF (2013). Chromosome pairing and synapsis during Caenorhabditis elegans meiosis. Curr. Opin. Cell Biol..

[8] Woglar A, Jantsch V (2014). Chromosome movement in meiosis I prophase of Caenorhabditis elegans. Chromosoma.

[9] Yu Z, Kim Y, Dernburg AF (2016). Meiotic recombination and the crossover assurance checkpoint in Caenorhabditis elegans. Semin. Cell Developmental Biol..

[10] Zetka M (2009). Homologue pairing, recombination and segregation in Caenorhabditis elegans. Genome Dyn..

[11] Zetka M, Rose A (1995). The genetics of meiosis in Caenorhabditis elegans. Trends Genet..

[12] Hillers, K. J., Jantsch, V., Martinez-Perez, E. & Yanowitz, J. L. Meiosis. *WormBook* **2017**, 1–43 (2017). 10.1895/wormbook.1.178.1 PMC5215044 26694509

[13] Checchi PM, Engebrecht J (2011). Heteromorphic sex chromosomes: navigating meiosis without a homologous partner. Mol. Reprod. Dev..

[14] Strome, S., Kelly, W. G., Ercan, S. & Lieb, J. D. Regulation of the X chromosomes in Caenorhabditis elegans. *Cold Spring Harbor Perspect. Biol.* **6**, ao18366 (2014). 10.1101/cshperspect.a018366 PMC3942922 24591522

[15] Maine EM (2010). Meiotic silencing in Caenorhabditis elegans. Int. Rev. Cell Mol. Biol..

[16] Vibranovski MD (2014). Meiotic sex chromosome inactivation in Drosophila. J. Genomics.

[17] Turner JM (2015). Meiotic silencing in mammals. Annu. Rev. Genet.

[18] Carofiglio F (2013). SPO11-independent DNA repair foci and their role in meiotic silencing. PLoS Genet.

[19] Becherel OJ (2013). Senataxin plays an essential role with DNA damage response proteins in meiotic recombination and gene silencing. PLoS Genet..

[20] Broering TJ (2014). BRCA1 establishes DNA damage signaling and pericentric heterochromatin of the X chromosome in male meiosis. J. Cell Biol..

[21] ElInati E (2017). DNA damage response protein TOPBP1 regulates X chromosome silencing in the mammalian germ line. Proc. Natl Acad. Sci. USA.

[22] Ichijima Y (2011). MDC1 directs chromosome-wide silencing of the sex chromosomes in male germ cells. Genes Dev..

[23] Hirota T (2018). SETDB1 links the meiotic DNA damage response to sex chromosome silencing in mice. Dev. Cell.

[24] Kouznetsova A (2009). BRCA1-mediated chromatin silencing is limited to oocytes with a small number of asynapsed chromosomes. J. Cell Sci..

[25] Mahadevaiah SK (2008). Extensive meiotic asynapsis in mice antagonises meiotic silencing of unsynapsed chromatin and consequently disrupts meiotic sex chromosome inactivation. J. Cell Biol..

[26] Kelly WG (2002). X-chromosome silencing in the germline of C. elegans. Development.

[27] Tzur YB (2018). Spatiotemporal gene expression analysis of the Caenorhabditis elegans germline uncovers a syncytial expression switch. Genetics.

[28] Bender LB (2006). MES-4: an autosome-associated histone methyltransferase that participates in silencing the X chromosomes in the C. elegans germ line. Development.

[29] Ebbing A (2018). Spatial transcriptomics of C. elegans males and hermaphrodites identifies sex-specific differences in gene expression patterns. Dev. Cell.

[30] Gaydos LJ, Rechtsteiner A, Egelhofer TA, Carroll CR, Strome S (2012). Antagonism between MES-4 and Polycomb repressive complex 2 promotes appropriate gene expression in C. elegans germ cells. Cell Rep..

[31] Turner JM (2005). Silencing of unsynapsed meiotic chromosomes in the mouse. Nat. Genet.

[32] Shiu PK, Raju NB, Zickler D, Metzenberg RL (2001). Meiotic silencing by unpaired DNA. Cell.

[33] Bean CJ, Schaner CE, Kelly WG (2004). Meiotic pairing and imprinted X chromatin assembly in Caenorhabditis elegans. Nat. Genet..

[34] Royo H (2010). Evidence that meiotic sex chromosome inactivation is essential for male fertility. Curr. Biol..

[35] Pinton A (2008). Meiotic studies in an azoospermic boar carrying a Y;14 translocation. Cytogenet. Genome Res..

[36] Barasc H (2012). Y-autosome translocation interferes with meiotic sex inactivation and expression of autosomal genes: a case study in the pig. Sex Dev..

[37] Vozdova M (2016). Meiotic behaviour of evolutionary sex-autosome translocations in Bovidae. Chromosome Res..

[38] Turner JM, Mahadevaiah SK, Ellis PJ, Mitchell MJ, Burgoyne PS (2006). Pachytene asynapsis drives meiotic sex chromosome inactivation and leads to substantial postmeiotic repression in spermatids. Dev. Cell.

[39] Mary, N. et al. Meiotic synapsis and gene expression altered by a balanced Y-autosome reciprocal translocation in an Azoospermic pig. *Sex. Dev.* **12**, 256–263 (2018). 10.1159/000491804 30179878

[40] Moller HD (2018). CRISPR-C: circularization of genes and chromosome by CRISPR in human cells. Nucleic Acids Res..

[41] Chen X (2018). Targeted chromosomal rearrangements via combinatorial use of CRISPR/Cas9 and Cre/LoxP technologies in Caenorhabditis elegans. G3.

[42] Chen X, Li M, Feng X, Guang S (2015). Targeted chromosomal translocations and essential gene knockout using CRISPR/Cas9 technology in Caenorhabditis elegans. Genetics.

[43] Cullot G (2019). CRISPR-Cas9 genome editing induces megabase-scale chromosomal truncations. Nat. Commun..

[44] Owens DDG (2019). Microhomologies are prevalent at Cas9-induced larger deletions. Nucleic Acids Res..

[45] Essletzbichler P (2014). Megabase-scale deletion using CRISPR/Cas9 to generate a fully haploid human cell line. Genome Res..

[46] Korablev AN, Serova IA, Serov OL (2017). Generation of megabase-scale deletions, inversions and duplications involving the Contactin-6 gene in mice by CRISPR/Cas9 technology. BMC Genet.

[47] Guilherme RS (2011). Mechanisms of ring chromosome formation, ring instability and clinical consequences. BMC Med. Genet.

[48] Takagaki N (2020). The mechanoreceptor DEG-1 regulates cold tolerance in Caenorhabditis elegans. EMBO Rep..

[49] Wang Y (2008). A glial DEG/ENaC channel functions with neuronal channel DEG-1 to mediate specific sensory functions in C. elegans. EMBO J..

[50] Ishtayeh H (2021). Systematic analysis of long intergenic non-coding RNAs in C. elegans germline uncovers roles in somatic growth. RNA Biol..

[51] Achache H (2019). Progression of meiosis is coordinated by the level and location of MAPK activation via OGR-2 in Caenorhabditis elegans. Genetics.

[52] Friedland AE (2013). Heritable genome editing in C. elegans via a CRISPR-Cas9 system. Nat. Methods.

[53] Phillips CM (2005). HIM-8 binds to the X chromosome pairing center and mediates chromosome-specific meiotic synapsis. Cell.

[54] Paulsen T, Kumar P, Koseoglu MM, Dutta A (2018). Discoveries of extrachromosomal circles of DNA in normal and tumor cells. Trends Genet..

[55] Traverse KL, Pardue ML (1988). A spontaneously opened ring chromosome of Drosophila melanogaster has acquired He-T DNA sequences at both new telomeres. Proc. Natl Acad. Sci. USA.

[56] Diag A, Schilling M, Klironomos F, Ayoub S, Rajewsky N (2018). Spatiotemporal m(i)RNA architecture and 3′ UTR regulation in the C. elegans germline. Dev. Cell.

[57] Bender LB, Cao R, Zhang Y, Strome S (2004). The MES-2/MES-3/MES-6 complex and regulation of histone H3 methylation in C. elegans. Curr. Biol..

[58] Schaner, C. E. & Kelly, W. G. Germline chromatin. *WormBook* 1–14, 10.1895/wormbook.1.73.1 (2006). 10.1895/wormbook.1.73.1 PMC4781259 18050477

[59] Reuben M, Lin R (2002). Germline X chromosomes exhibit contrasting patterns of histone H3 methylation in Caenorhabditis elegans. Dev. Biol..

[60] Vielle A (2012). H4K20me1 contributes to downregulation of X-linked genes for C. elegans dosage compensation. PLoS Genet.

[61] Furuhashi H (2010). Trans-generational epigenetic regulation of C. elegans primordial germ cells. Epigenetics Chromatin.

[62] Rechtsteiner A (2010). The histone H3K36 methyltransferase MES-4 acts epigenetically to transmit the memory of germline gene expression to progeny. PLoS Genet..

[63] Seydoux G, Dunn MA (1997). Transcriptionally repressed germ cells lack a subpopulation of phosphorylated RNA polymerase II in early embryos of Caenorhabditis elegans and Drosophila melanogaster. Development.

[64] Kim E, Du L, Bregman DB, Warren SL (1997). Splicing factors associate with hyperphosphorylated RNA polymerase II in the absence of pre-mRNA. J. Cell Biol..

[65] Nousch M, Eckmann CR (2013). Translational control in the Caenorhabditis elegans germ line. Adv. Exp. Med. Biol..

[66] Letourneau A (2014). Domains of genome-wide gene expression dysregulation in Down’s syndrome. Nature.

[67] Pelleri MC (2018). Integrated quantitative transcriptome maps of human trisomy 21 tissues and cells. Front. Genet..

[68] Hodgkin J, Horvitz HR, Brenner S (1979). Nondisjunction mutants of the nematode CAENORHABDITIS ELEGANS. Genetics.

[69] Colaiacovo MP (2003). Synaptonemal complex assembly in C. elegans is dispensable for loading strand-exchange proteins but critical for proper completion of recombination. Dev. Cell.

[70] Alpi A, Pasierbek P, Gartner A, Loidl J (2003). Genetic and cytological characterization of the recombination protein RAD-51 in Caenorhabditis elegans. Chromosoma.

[71] Rinaldo C, Bazzicalupo P, Ederle S, Hilliard M, La Volpe A (2002). Roles for Caenorhabditis elegans rad-51 in meiosis and in resistance to ionizing radiation during development. Genetics.

[72] Tzur YB (2013). Heritable custom genomic modifications in Caenorhabditis elegans via a CRISPR-Cas9 System. Genetics.

[73] Chiu H, Schwartz HT, Antoshechkin I, Sternberg PW (2013). Transgene-free genome editing in Caenorhabditis elegans using CRISPR-Cas. Genetics.

[74] Cho SW, Kim S, Kim JM, Kim JS (2013). Targeted genome engineering in human cells with the Cas9 RNA-guided endonuclease. Nat. Biotechnol..

[75] Dickinson DJ, Ward JD, Reiner DJ, Goldstein B (2013). Engineering the Caenorhabditis elegans genome using Cas9-triggered homologous recombination. Nat. Methods.

[76] Farboud B, Meyer BJ (2015). Dramatic enhancement of genome editing by CRISPR/Cas9 through improved guide RNA design. Genetics.

[77] Katic I, Grosshans H (2013). Targeted heritable mutation and gene conversion by Cas9-CRISPR in Caenorhabditis elegans. Genetics.

[78] Lo TW (2013). Precise and heritable genome editing in evolutionarily diverse nematodes using TALENs and CRISPR/Cas9 to engineer insertions and deletions. Genetics.

[79] Paix, A., Folkmann, A., Rasoloson, D. & Seydoux, G. High efficiency, homology-directed genome editing in Caenorhabditis elegans using CRISPR/Cas9 ribonucleoprotein complexes. *Genetics* **201**, 47–54 (2015). 10.1534/genetics.115.179382 PMC4566275 26187122

[80] Paix A (2014). Scalable and versatile genome editing using linear DNAs with microhomology to Cas9 sites in Caenorhabditis elegans. Genetics.

[81] Herman RK, Kari CK, Hartman PS (1982). Dominant X-chromosome nondisjunction mutants of Caenorhabditis elegans. Genetics.

[82] MacQueen AJ (2005). Chromosome sites play dual roles to establish homologous synapsis during meiosis in C. elegans. Cell.

[83] Smolikov S, Schild-Prufert K, Colaiacovo MP (2009). A yeast two-hybrid screen for SYP-3 interactors identifies SYP-4, a component required for synaptonemal complex assembly and chiasma formation in Caenorhabditis elegans meiosis. PLoS Genet.

[84] Clark DV, Rogalski TM, Donati LM, Baillie DL (1988). The unc-22(IV) region of Caenorhabditis elegans: genetic analysis of lethal mutations. Genetics.

[85] Ferguson EL, Horvitz HR (1985). Identification and characterization of 22 genes that affect the vulval cell lineages of the nematode Caenorhabditis elegans. Genetics.

[86] Li XY, Gui JF (2018). Diverse and variable sex determination mechanisms in vertebrates. Sci. China Life Sci..

[87] Wilson Sayres MA (2018). Genetic diversity on the sex chromosomes. Genome Biol. evolution.

[88] Richardson LA (2016). Sex chromosomes do it differently. PLoS Biol..

[89] Petropoulos S (2016). Single-cell RNA-seq reveals lineage and X chromosome dynamics in human preimplantation embryos. Cell.

[90] McClendon TB (2016). Chromosome crossover formation and genome stability in Caenorhabditis elegans are independently regulated by xnd-1. G3.

[91] Wagner CR, Kuervers L, Baillie DL, Yanowitz JL (2010). xnd-1 regulates the global recombination landscape in Caenorhabditis elegans. Nature.

[92] Jaramillo-Lambert A, Ellefson M, Villeneuve AM, Engebrecht J (2007). Differential timing of S phases, X chromosome replication, and meiotic prophase in the C. elegans germ line. Dev. Biol..

[93] Lamelza P, Bhalla N (2012). Histone methyltransferases MES-4 and MET-1 promote meiotic checkpoint activation in Caenorhabditis elegans. PLoS Genet..

[94] Meneely PM, Farago AF, Kauffman TM (2002). Crossover distribution and high interference for both the X chromosome and an autosome during oogenesis and spermatogenesis in Caenorhabditis elegans. Genetics.

[95] Mlynarczyk-Evans S, Villeneuve AM (2017). Time-course analysis of early meiotic prophase events informs mechanisms of homolog pairing and synapsis in Caenorhabditis elegans. Genetics.

[96] Guo Y, Yang B, Li Y, Xu X, Maine EM (2015). Enrichment of H3K9me2 on unsynapsed chromatin in Caenorhabditis elegans does not target de novo sites. G3.

[97] Libuda DE, Uzawa S, Meyer BJ, Villeneuve AM (2013). Meiotic chromosome structures constrain and respond to designation of crossover sites. Nature.

[98] Mets DG, Meyer BJ (2009). Condensins regulate meiotic DNA break distribution, thus crossover frequency, by controlling chromosome structure. Cell.

[99] Murakami H (2020). Multilayered mechanisms ensure that short chromosomes recombine in meiosis. Nature.

[100] Brenner S (1974). The genetics of Caenorhabditis elegans. Genetics.

[101] Saito TT, Youds JL, Boulton SJ, Colaiacovo MP (2009). Caenorhabditis elegans HIM-18/SLX-4 interacts with SLX-1 and XPF-1 and maintains genomic integrity in the germline by processing recombination intermediates. PLoS Genet.

[102] Lanctot C, Meister P (2013). Microscopic analysis of chromatin localization and dynamics in C. elegans. Methods Mol. Biol..

[103] Edgar LG (1995). Blastomere culture and analysis. Methods Cell Biol..

[104] Li H (2018). Minimap2: pairwise alignment for nucleotide sequences. Bioinformatics.

[105] Sedlazeck FJ (2018). Accurate detection of complex structural variations using single-molecule sequencing. Nat. Methods.

[106] Cretu Stancu M (2017). Mapping and phasing of structural variation in patient genomes using nanopore sequencing. Nat. Commun..

[107] Heller D, Vingron M (2019). SVIM: structural variant identification using mapped long reads. Bioinformatics.

[108] Scheinin I (2014). DNA copy number analysis of fresh and formalin-fixed specimens by shallow whole-genome sequencing with identification and exclusion of problematic regions in the genome assembly. Genome Res..

[109] Li H (2009). The sequence alignment/Map format and SAMtools. Bioinformatics.

[110] Poplin R (2018). A universal SNP and small-indel variant caller using deep neural networks. Nat. Biotechnol..

[111] McLaren W (2016). The Ensembl variant effect predictor. Genome Biol..

[112] Govindan JA, Cheng H, Harris JE, Greenstein D (2006). Galphao/i and Galphas signaling function in parallel with the MSP/Eph receptor to control meiotic diapause in C. elegans. Curr. Biol..

[113] Govindan JA, Nadarajan S, Kim S, Starich TA, Greenstein D (2009). Somatic cAMP signaling regulates MSP-dependent oocyte growth and meiotic maturation in C. elegans. Development.

[114] Rual JF (2004). Toward improving Caenorhabditis elegans phenome mapping with an ORFeome-based RNAi library. Genome Res.

