# Supplementary material for: Bisection of the X chromosome disrupts the initiation of chromosome silencing during meiosis in Caenorhabditis elegans
Source: Nat Commun. 2021 Aug 10;12:4802. doi: 10.1038/s41467-021-24815-0 (PMC8355143; doi:10.1038/s41467-021-24815-0)
Supplement: Supplementary file 1 — Supplementary figures and tables [file 41467_2021_24815_MOESM1_ESM.pdf]

Supplementary Information

Bisection of the X Chromosome Disrupts the Initiation of Chromosome Silencing during Meiosis in *Caenorhabditis elegans*

Yisrael Rappaport, Hanna Achache, Roni Falk, Omer Murik, Oren Ram, & Yonatan B. Tzur

Supplementary Figure 1

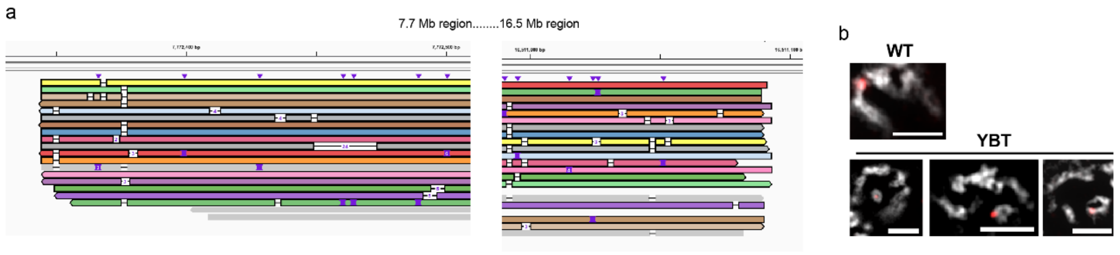

Supplementary Figure 1: The internal segment of YBT7 may exist as a ring. a, IGV[1] screen captures of Nanopore genome sequencing data of YBT7. Individual reads are color coded. Note that the same reads continue with the same orientation between the two genomic regions. **b,** Late pachytene nuclei stained with DAPI (white) and the FISH probe marking a site left of *linc-20* (red). n=105 nuclei. Scale bars = 3 μM.

1 Robinson, J. T. et al. Integrative genomics viewer. *Nat. Biotechnol*. **29**, 24–26 (2011).

Supplementary Figure 2

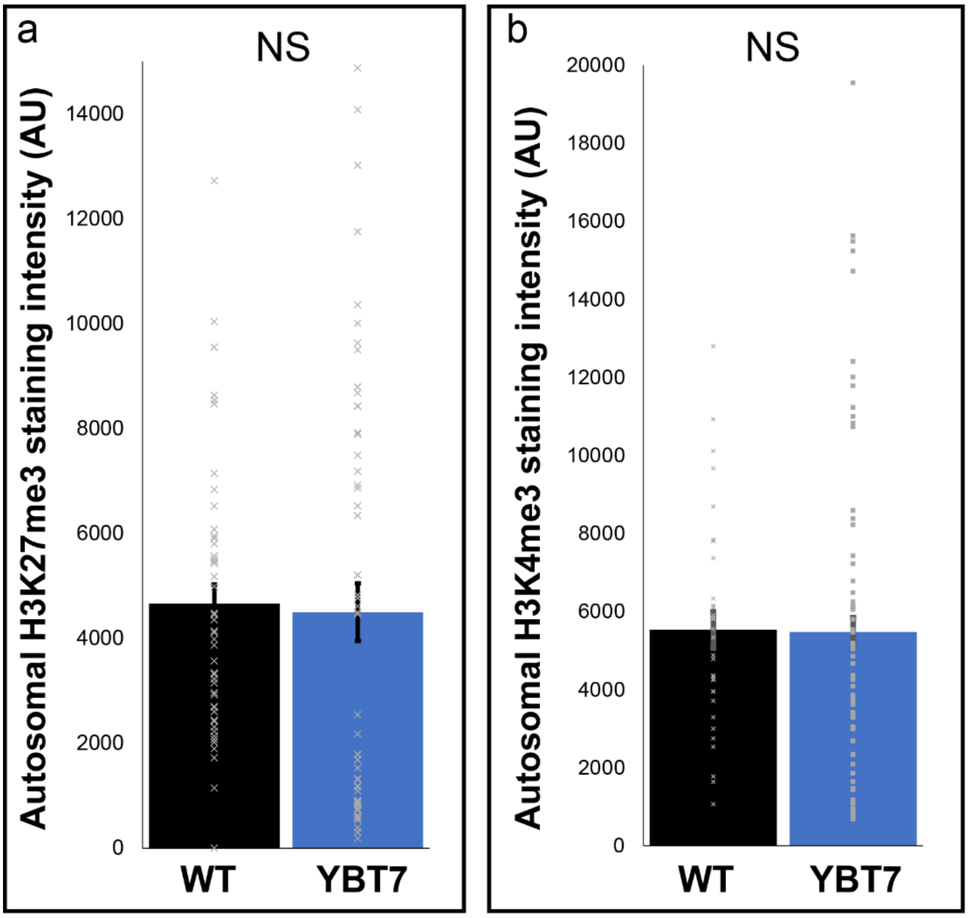

Supplementary Figure 2: The level of histone marks on autosomes is not significantly different in YBT7 and wild-type gonads. Averages ± SEM of **a,** H3K27me3 and **b,** H3K4me3 signals on autosomes of wild-type and YBT7 gonads. n=47, 55 nuclei in a and 33, 66 nuclei in b for WT and YBT7 respectively. NS: Not significant. *p* value = 0.07 and 0.14457 by the one-tailed Mann-Whitney test for a and b respectively.

Supplementary Figure 3

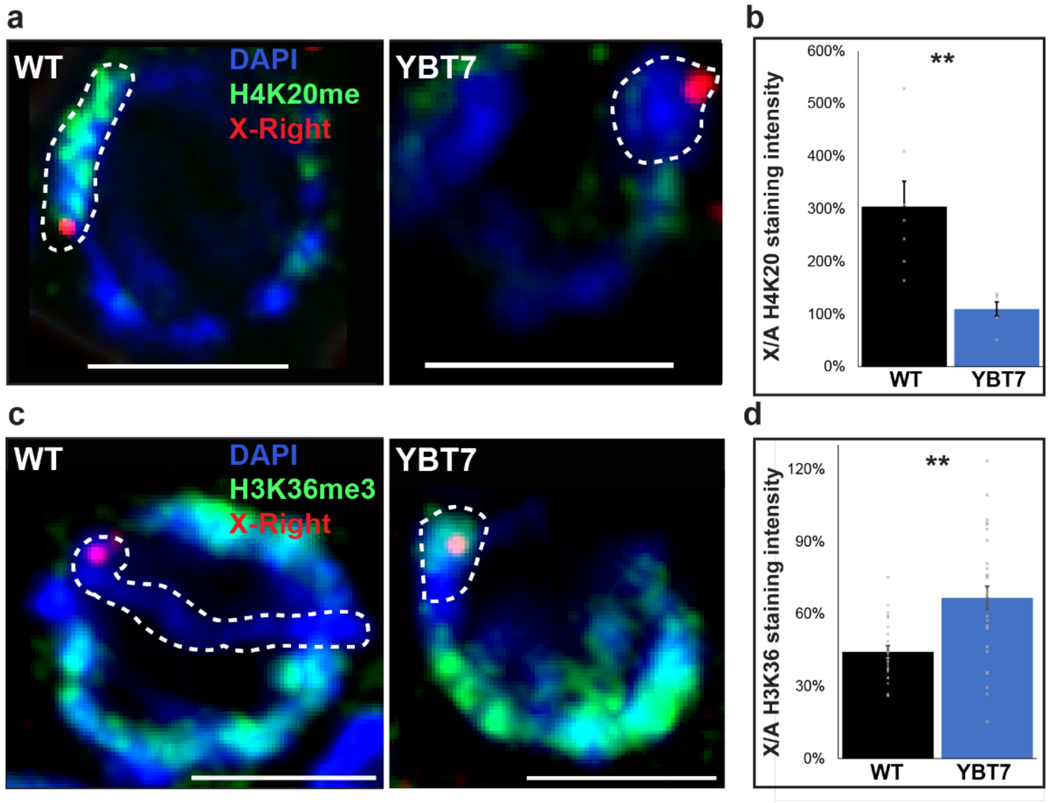

Supplementary Figure 3: Active transcription marks are associated with segments of the X chromosome in YBT7. a, Mid-late-pachytene nuclei stained with DAPI (blue), X-right FISH probe (red), and antibody against H4K20me (green). **b,** Averages ± SEM of the relative H4K20me signals in wild-type and YBT7 nuclei on X-right marked bodies vs. an autosome. n=7 and 6 nuclei for WT and YBT7 respectively. *p* value = 0.00338 by the two-tailed Mann-Whitney test. **c,** Mid-pachytene nuclei stained with DAPI (blue), X-right FISH probe (red), and antibody against H3K36me3 (green). **d,** Averages ± SEM of the relative H3K36me3 signals in wild-type and YBT7 nuclei on X-right -marked bodies vs. autosomes. n=24 and 29 nuclei for WT and YBT7 respectively. *p* value = 0.00112 by the two-tailed Mann-Whitney test. ** p<0.01, by the two-tailed Mann-Whitney test. Scale bar = 3 μM. Dotted lines: FISH marked body.

Supplementary Figure 4

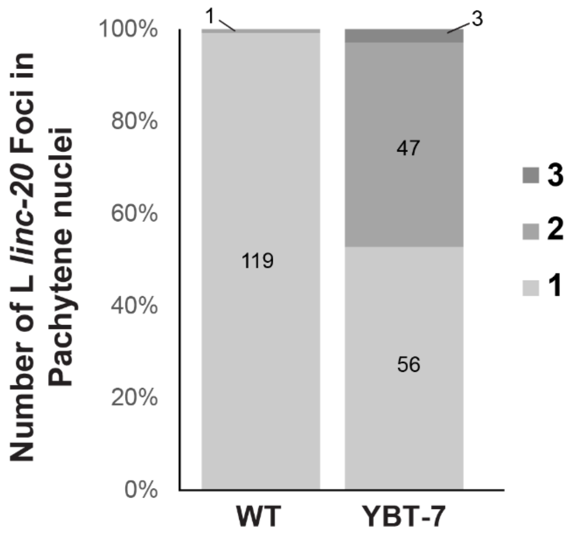

Supplementary Figure 4: The number of internal segments varies in YBT7. Distribution of the percentages of the DAPI-stained bodies marked by L-*linc-20* FISH signal in wild-type and YBT7 pachytene nuclei. Numbers on bars represent the number of scored oocytes.

Supplementary Figure 5

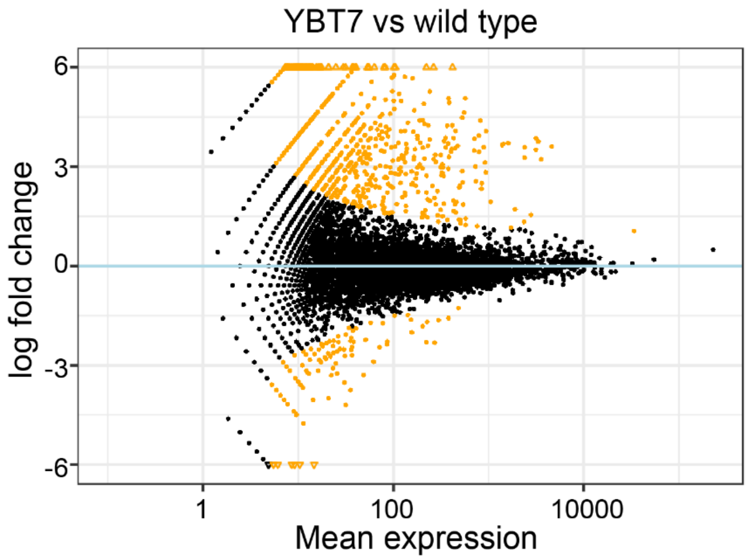

Supplementary Figure 5: Differential gene expression in YBT7 and wild-type gonads. Expression differences in YBT7 vs. wild-type gonads for each gene plotted against the average expression. The x axis is the DESeq2-calculated baseMean, and the y axis is the DESeq2-calculated lfcMLE (log2 of the fold-change maximum likelihood estimate). Orange dots indicate differentially expressed genes, other genes are indicated as black dots. The y axis was limited to a range between -6 and 6; triangles represent genes with lfcMLE value beyond this range.

Supplementary Figure 6

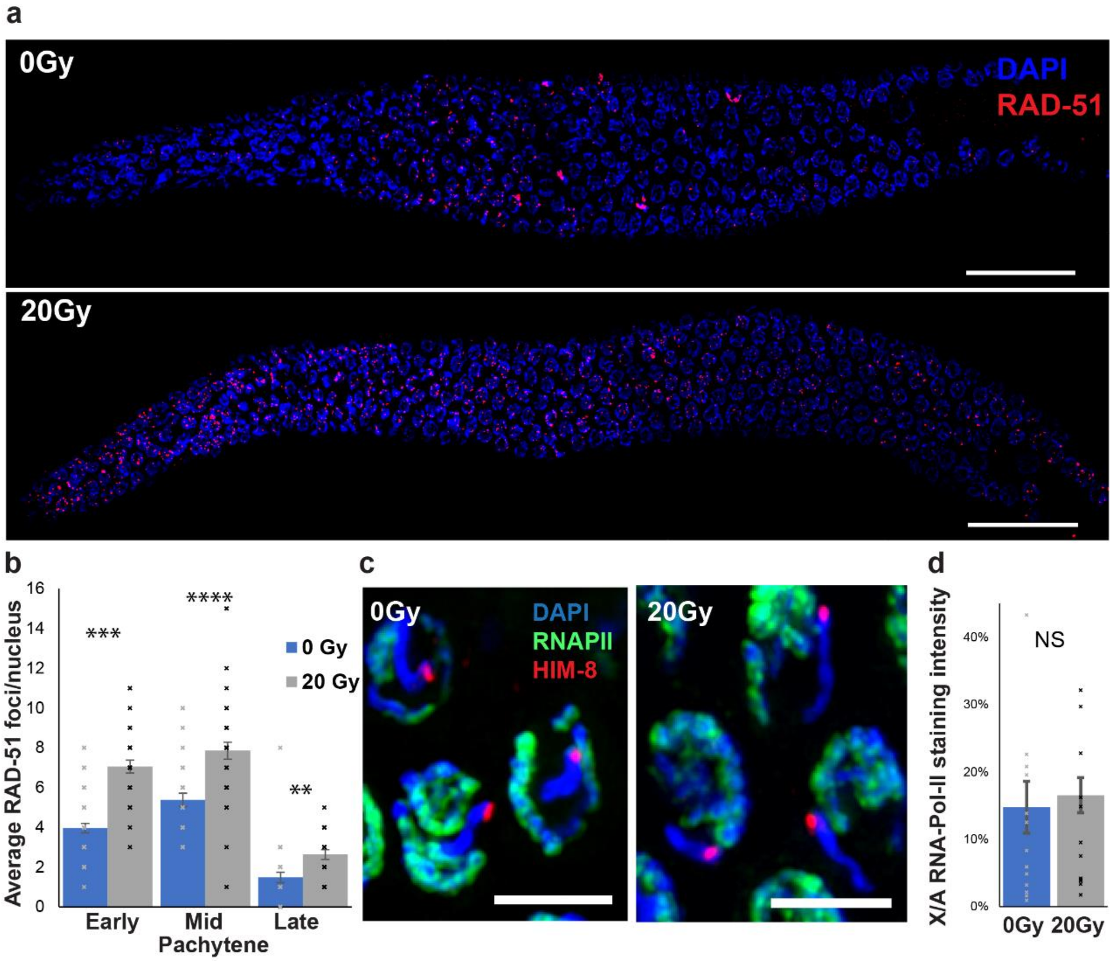

Supplementary Figure 6: Increases in DNA double-strand breaks do not reduce X chromosome silencing. a, Whole-mount gonads stained with DAPI (blue) and anti-RAD-51 (red) following 0 or 20 Gy ionizing radiation. Scale bar = 50 μM. **b,** Average ± SEM RAD-51 foci per nucleus during pachytene. n=45, 32, 34 at 0 Gy and 31, 31 and 25 at 20 Gy for early mid- and late pachytene nuclei respectively. **c,** Mid-pachytene nuclei of control and irradiated worms stained with DAPI (blue), anti-RNAPII (green), and anti-HIM-8 (red). Scale bar = 3 μM. **d,** Averages ± SEM of the relative RNAPII signal per control or irradiated nucleus on HIM-8-marked body vs. an autosome. n=23 and 20 nuclei for 0 Gy and 20 Gy respectively. *p* value = 0.08076 by the one-tailed Mann-

Whitney test. ** $p<0.01$, *** $p<0.001$, **** $p<0.0001$, by the two-tailed Mann-Whitney test. N.S. indicates not significant.

Supplementary Figure 7

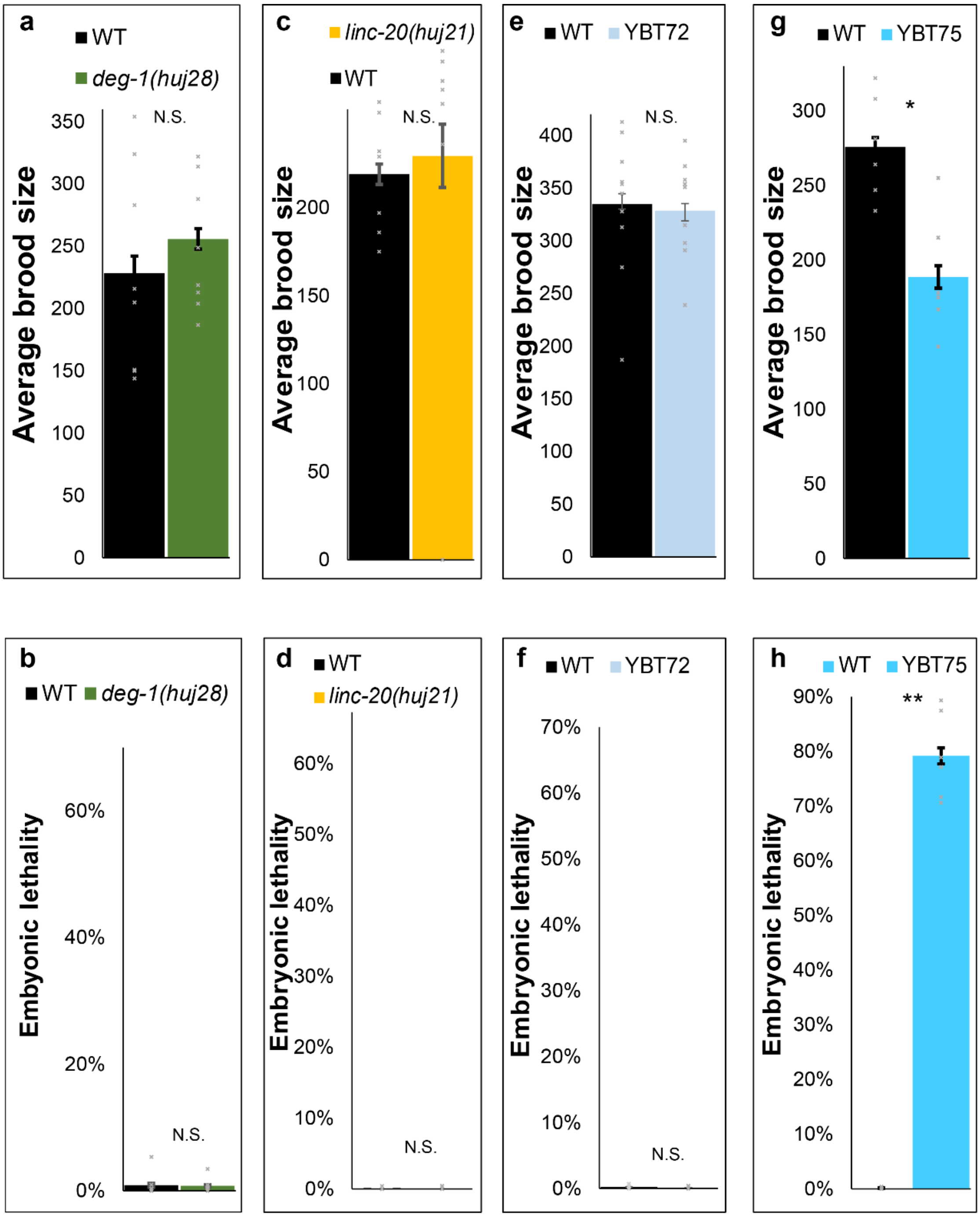

Supplementary Figure 7: Meiotic defects in YBT7 are the result of the chromosome bisection and not local gene expression changes. a, Average ± SEM progeny brood size per worm and **b,** embryonic lethality of wild type vs. *deg-1(huj28)* (disruption within *deg-1*). n=8 and 9 worms for WT and *deg-1(huj28)* respectively. **c,** Average ± SEM progeny brood size per worm and **d,** embryonic lethality of wild type vs. *linc-20(huj21)* (full deletion of *linc-20*) n=7

worms. **e,** Average ± SEM progeny brood size per worm and **f,** embryonic lethality of wild type vs. YBT72 (deletions within *deg-1* and *linc-20,* identical to YBT7). n=10 worms. **g,** Average ± SEM progeny brood size per worm and **h,** embryonic lethality of wild type vs. YBT75 (YBT68 with reconstructed wild-type *deg-1*) n=6 worms. *p*-value is 0.01314 and 0.00512 for g and h respectively, by the two-tailed Mann-Whitney test. * $p<0.05$, ** $p<0.01$, N.S. indicates not significant, by the two-tailed Mann-Whitney test.

Supplementary Figure 8

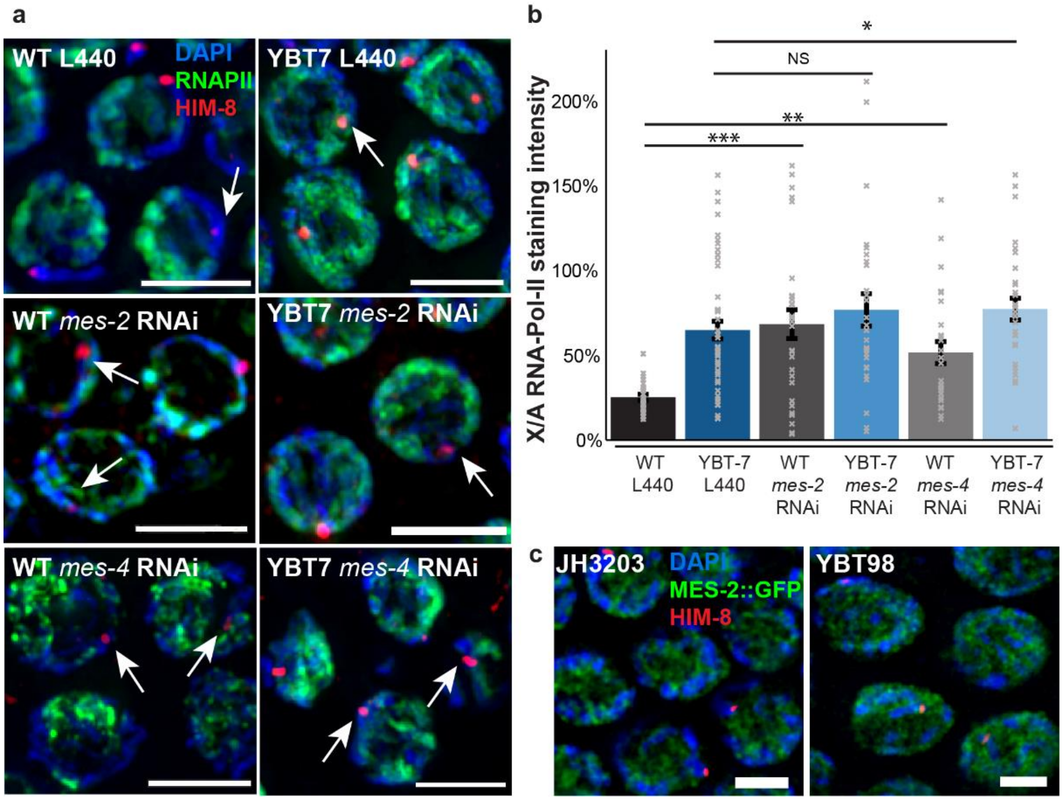

Supplementary Figure 8: Interaction of YBT7 with *mes* genes. a, Mid-pachytene nuclei stained with DAPI (blue), anti-RNAPII (green), and anti-HIM-8 (red). Arrows indicate HIM-8-marked bodies. **b,** Averages ± SEM of the relative RNAPII signals per nuclei on HIM-8-marked bodies vs. autosomes. n=25, 51, 29, 27, 26, and 30 nuclei for wild-type, YBT7, *mes-2* RNAi, YBT7 *mes-2* RNAi, *mes-4* RNAi and YBT7 *mes-4* RNAi respectively. **c,** Mid-pachytene nuclei of JH3203 and YBT98, which express GFP::MES-2 (green), stained with DAPI (blue) and anti-HIM-8 (red). n=30 nuclei. Scale bar = 3 μM**.** * *p*=0.04363 by the one-tailed Mann-Whitney test, ** *p*=0.00116, ***=0.00036 by the two-tailed Mann-Whitney test. N.S. indicates not significant.

Supplementary Figure 9

Figure S9

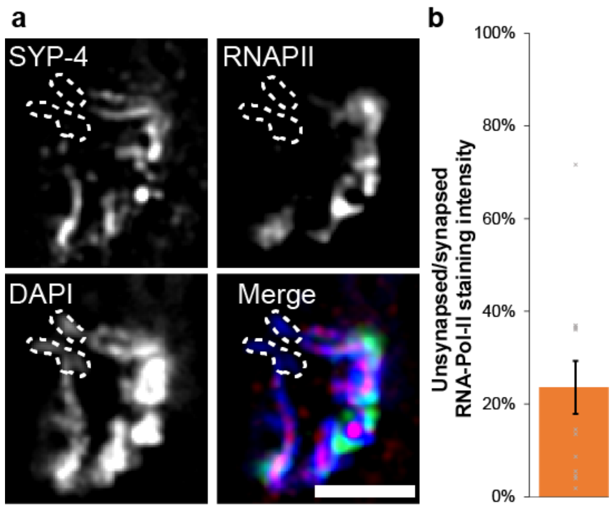

Supplementary Figure 9: Segmented autosomes undergo MSUC. a, Mid-pachytene nT1/+ nuclei stained with DAPI (blue), anti-RNAPII (green), and anti-SYP-4 (red). Dotted lines indicate unsynapsed tracks. Scale bar = 3 μM. **b,** Averages ± SEM of the relative RNAPII signal on an unsynapsed track vs. a synapsed track. n=13.

Supplementary Table 1

YBT7

CHROM	POS	TYPE	REF	ALT	gene
III	346631	indel	A	[ATAAATATTTAGCAGACCAAAGTTGGGTATGCTAAATATTTAGCAGACCAAAGTTGGGTATGCTAAATATTTAGCAGACCAAAGTTGGGTATGCTAAATATTTAGCAGACCAAAGTTGGGTATGC]	gei-4
IV	2524056	snp	A	[T]	Y69A2AR.16
V	20240213	indel	ATTTTGGGCGCTGCTTAGACTACAAACTACAAG	[A]	swsn-1

YBT68

CHROM	POS	TYPE	REF	ALT	gene
I	955230	indel	CTA	[C]	mig-1
III	6071174	snp	A	[T]	C05D10.2
III	10836218	indel	TC	[T]	R17.3
III	12337767	indel	GTATATATATATATATATATATATATATATATATATATATATATATATATATATATATATATATATATATA	[G]	Y75B8A.55
V	11119315	snp	G	[T]	H19N07.3
V	11690781	indel	A	[AAAAATTTTCAATAAAAAATCAAATAAATAAATAAATAAATATGCTGATAAAAACACACAAACTATTGAATTGATATCACTGATAAAATAATAAAGAATAATCAGTCTTGCTTTGGAATTTTTATTCAAAAATCAAGACTGATTATTCTTTATTATTTTATCAGTGATATCAATTCAATAGTTTGTGT	W05E10.1

				GTTTTTATCAGCAT ATTTATTTATTTAT TTATTTGATTTTTT ATTGAAAATTTTTT ATTG]	
V	14785 254	indel	TGGAACTCCTGGGA ATGTAGCATATCTC ACACATCCAACAGC CTCGTCCGGTACG GCAATCTTCACAAA TGCGTCACACGTGG CCGCCGAAATCACT TGC	[T]	rin-1
V	19029 747	snp	G	[A]	Y39B6A.83
X	77697 37	indel	GTTCATCCATCGTC TCAGCGCTGC	[G]	deg-1
X	16413 763	snp	G	[C]	eat-17

Supplementary Table 1: Point mutations in YBT7 and YBT68. Homozygous mutations detected by Illumina sequencing in YBT7 and YBT68 but not in the parental wild-type strain.

Supplementary Table 2

Name	Sequence	Description	Strains used
homologous lincs sgRNA 5A	GTCGAGCAGCGCTGAGACGA	Cut 5' *of linc-20* and deg-1 intron 14	YBT7
homologous lincs sgRNA 5B	GAAATTCGCTTCAGTTTTCA	Cut 5' *of linc-20* and deg-1 intron 14	YBT7
homologous lincs sgRNA 3A	GCGAACAAACTCGAGCATAA	Cut 3' *of linc-20* and deg-1 intron 14	YBT7
homologous lincs sgRNA 3B	AAGTTCGCGCGTGCTTCGAA	Cut 3' *of linc-20* and deg-1 intron 14	YBT7
homologous lincs 5' crRNA	GTCGAGCAGCGCTGAGACGA	Cut 5' *of linc-20* and deg-1 intron 14	YBT68, YBT54, YBT72
homologous lincs 3' crRNA	GCGAACAAACTCGAGCATAA	Cut 3' *of linc-20* and deg-1 intron 14	, YBT68, YBT54, YBT72
Linc-20 del1-ssODN	GGAGCAAAATGCTCGTCTTTGCGAACAAACT CGAGCACAGCGCTGCTCGACACGGCGGTCG GTGACTGTTCGGC	Template for creating *linc-20* deletion identical to YBT7	YBT72
Linc-20 YBT7 del-2 5' crRNA	AACTGTTTTTGTTGGCTTAG	Cut 5' *of linc-20* upstream location for mutagenesis	YBT72
Linc-20 YBT7 del-2 3' crRNA	AAAACAACTTTGTAGTTGAC	Cut 3' *of linc-20* upstream location for mutagenesis	YBT72
Linc-20 del2-ssODN	TTATCTCGACCAAAAAATTTTTTGACAAAAAT CGGTCAACTACAAAGTTGTTTTTTAATTAGAA ATGCACATCGAATTACGTCATACACTTTGATG AACTGTTTTTGTTGGCTTAGAGAATTAGGCTG TGTTTTACAATTATAATGTCAT	Template for creating *linc-20* upstream deletion identical to YBT7	YBT72
Deg-1 YBT7 del- 5' crRNA	GTCGAGCAGCGCTGAGACGA	Cut 5' *deg-1* i-14 location for mutagenesis	YBT72
Deg-1 YBT7 del- 3' crRNA	TTGATGAAAATCATTTTTTT	Cut 3' *deg-1* i-14 location for mutagenesis	YBT72
Deg-1 del-ssODN	CTGAAGCGAATTTCCAATATCGCTGTGTTCAT CCATCACTACAAAAAATCAAATTAAAAATTTTT TCGAAAAAAATGATTTTCATCAATTTTTCCATT TCAGTGTTA	Template for creating *deg-1* i-14 deletion identical to YBT7	YBT72
YBT68 deg-1 crRNA	CCAATATCGCTGTGTCGACA	Cut upstream the *deg-1* i-14	YBT75

		mutation in YBT68	
Deg-1-i14-YBT68toN2-ssODN	CTTGAAAACTGAAGCGAATTTCCAATATCGCTGTGTTCATCCATCGTCTCAGCGCTGCTCGACACGGCGGTCGGTGACTGTTCGGCAATCTTAAAT	Template for fixing deg-1 i14 mutation in YBT68	YBT75
Deg-1 exon-1 crRNA	GAATTTGAAAATCAACTTGC	crRNA for creating a mutation in the beginning of *deg-1*	YBT67

Supplementary Table 2: List of gRNAs, crRNAs, and single-stranded oligonucleotides used for genome engineering.
