# Supplementary material for: Bisection of the X chromosome disrupts the initiation of chromosome silencing during meiosis in Caenorhabditis elegans
Source: Nat Commun. 2021 Aug 10;12:4802. doi: 10.1038/s41467-021-24815-0 (PMC8355143; doi:10.1038/s41467-021-24815-0)
Supplement: Supplementary file 3 — Description of additional supplementary files [file 41467_2021_24815_MOESM3_ESM.docx]

Description of additional supplementary information

Title: Supplementary data 1

Description: Differential expression in YBT7 versus wild-type gonads. Columns include general gene details (A-F), raw counts for each strain (G and H), normalized counts (I and J), the DESeq2 calculated baseMean (K), the DESeq2 calculated lfcMLE (L), and a column for the final decision of differential expression (M). Genes with less than 3 counts are indicated with question marks in columns I to M. There is a question mark in the general gene details when information was missing from the database. The final decision column (M) has the value ‘THR’ for differentially expressed genes (i.e., those with a baseMean greater than 5 and a lfcMLE greater than 5/baseMean^0.5 + 1) and a value ‘FALSE’ for all other genes.
