# Supplementary material for: Bisection of the X chromosome disrupts the initiation of chromosome silencing during meiosis in Caenorhabditis elegans
Source: Nat Commun. 2021 Aug 10;12:4802. doi: 10.1038/s41467-021-24815-0 (PMC8355143; doi:10.1038/s41467-021-24815-0)
Supplement: Supplementary file 4 — Reporting Summary [file 41467_2021_24815_MOESM4_ESM.pdf]

nature research

Corresponding author(s): Yonatan Tzur
Last updated by author(s): Jun 1, 2021

Reporting Summary

Nature Research wishes to improve the reproducibility of the work that we publish. This form provides structure for consistency and transparency in reporting. For further information on Nature Research policies, see our Editorial Policies and the Editorial Policy Checklist.

Statistics

For all statistical analyses, confirm that the following items are present in the figure legend, table legend, main text, or Methods section.

n/a	Confirmed	
☐	☒	The exact sample size (*n*) for each experimental group/condition, given as a discrete number and unit of measurement
☐	☒	A statement on whether measurements were taken from distinct samples or whether the same sample was measured repeatedly
☐	☒	The statistical test(s) used AND whether they are one- or two-sided<br>*Only common tests should be described solely by name; describe more complex techniques in the Methods section.*
☒	☐	A description of all covariates tested
☐	☒	A description of any assumptions or corrections, such as tests of normality and adjustment for multiple comparisons
☐	☒	A full description of the statistical parameters including central tendency (e.g. means) or other basic estimates (e.g. regression coefficient) AND variation (e.g. standard deviation) or associated estimates of uncertainty (e.g. confidence intervals)
☐	☒	For null hypothesis testing, the test statistic (e.g. *F*, *t*, *r*) with confidence intervals, effect sizes, degrees of freedom and *P* value noted<br>*Give P values as exact values whenever suitable.*
☒	☐	For Bayesian analysis, information on the choice of priors and Markov chain Monte Carlo settings
☒	☐	For hierarchical and complex designs, identification of the appropriate level for tests and full reporting of outcomes
☒	☐	Estimates of effect sizes (e.g. Cohen's *d*, Pearson's *r*), indicating how they were calculated

Our web collection on statistics for biologists contains articles on many of the points above.

Software and code

Policy information about availability of computer code

Data collection	CellSense V. 1.8, FV10-ASW 3.1.
Data analysis	Autoquant X3, ImageJ 1.5P, Cutadapt 1.12, TopHat2 2.1.1, DESeq2 1.12.4, NanoFilt 2.6.0, NanoSV 1.2.3, SVIM 1.2.0, bwa-0.7.5a 100 mem Picard tools 769 v.2.8.1, GATK v3.7 101, Ensembl's Variant Effect Predictor v.83, sniffles version 1.0.11, , fastq quality filter FASTX version 0.0.14, minimap2, version 2.17, R package QDNAseq 3.5.2.

For manuscripts utilizing custom algorithms or software that are central to the research but not yet described in published literature, software must be made available to editors and reviewers. We strongly encourage code deposition in a community repository (e.g. GitHub). See the Nature Research guidelines for submitting code & software for further information.

Data

Policy information about availability of data

All manuscripts must include a data availability statement. This statement should provide the following information, where applicable:
- Accession codes, unique identifiers, or web links for publicly available datasets
- A list of figures that have associated raw data
- A description of any restrictions on data availability

Table S2 contains detailed descriptions of all primers used for genome engineering and genotyping. Source RNASeq data are provided at NCBI's Gene Expression Omnibus, under accession number GSE171938 [https://www.ncbi.nlm.nih.gov/geo/query/acc.cgi?acc=GSE171938].

Field-specific reporting

Please select the one below that is the best fit for your research. If you are not sure, read the appropriate sections before making your selection.

☒ Life sciences ☐ Behavioural & social sciences ☐ Ecological, evolutionary & environmental sciences

For a reference copy of the document with all sections, see nature.com/documents/nr-reporting-summary-flat.pdf

Life sciences study design

All studies must disclose on these points even when the disclosure is negative.

Sample size	Samples size were chosen based on the accepted publications in the field including number of individual repeats and worms/nuclei (e.g., Kelly et al 2002, Colaiacovo et al 2003). Statistical tests were made to verify these are appropriate.
Data exclusions	No data were excluded
Replication	Experiments were replicated several times. All replications successfully supported the conclusions.
Randomization	Not relevant since worms were picked randomly from NGM plates from all strains, and washed randomly from plates.
Blinding	Researchers were blinded while collecting the samples for the NGS. NGS data were analyze by blinded personal. Collection of IF and functional experiments could not be blinded due to visual differences from WT: NGM plates with high Emb phenotype and meiocytes with extra DAPI bodies. During analysis of all experiments the researchers were blinded.

Reporting for specific materials, systems and methods

We require information from authors about some types of materials, experimental systems and methods used in many studies. Here, indicate whether each material, system or method listed is relevant to your study. If you are not sure if a list item applies to your research, read the appropriate section before selecting a response.

Materials & experimental systems

n/a	Involved in the study	
☐	☒	Antibodies
☒	☐	Eukaryotic cell lines
☒	☐	Palaeontology and archaeology
☐	☒	Animals and other organisms
☒	☐	Human research participants
☒	☐	Clinical data
☒	☐	Dual use research of concern

Methods

n/a	Involved in the study	
☒	☐	ChIP-seq
☒	☐	Flow cytometry
☒	☐	MRI-based neuroimaging

Antibodies

Antibodies used	rabbit anti-SYP-4 (1:200, from S. Smolikove, The University of Iowa), goat anti-SYP-1 (1:200, from S. Smolikove, The University of Iowa), rabbit anti-HIM-8 (Novus Biological cat # 41980002, 1:2000), rat anti-HIM-8 (1:100, from A. Dernburg, University of California, Berkeley), rabbit anti-H3K27me3 (Millipore cat # 07-449, 1:1000), rabbit anti-H3K4me3 (Millipore cat # 05-745, 1:1000), mouse anti-H3K9me2 (Abcam cat # ab1220, 1/200), rabbit anti-H4K20me (Abcam cat # ab9051, 1/200), rabbit anti-H3K36me3 (Abcam cat # ab9050, 1/200), mouse anti-pSer2 RNAPII (Diagenode cat # C15200005, 1:1000). All secondary antibodies used were purchased from Jackson ImmunoResearch Laboratories, and used at 1/200 dilution: Cy2-donkey anti-rabbit (AB_2340612), Cy3-goat anti-rabbit (AB_2338000), Cy3-donkey anti-goat (AB_2307351), Cy2-goat anti-rat (AB_2338278), Cy2-goat anti-mouse (AB_2338746), Cy3-goat anti-mouse (AB_2338690), Cy5-donkey anti-mouse (AB_2338746), Cy5-donkey anti-rabbit (AB_2340607).
Validation	All secondary antibodies were tested for non specific binding and bleed through. SYP-1 and SYP-4 antibodies validation for C. elegans IF was described in Harrell et al 2021. HIM-8 antibody IF validation for C. elegans is described in Phillips et al 2005. Commercial antibodies were purchased based on validation statement on the manufacturer website, as well as from data presented in: McManus et al 2018, Jänes et al 2018, Gushchanskaia et al 2019, Pu et al 2018, Delaney et al 2019, Vielle et al 2012, Bessler et al 2012, Lee et al 2019, Lin et al 2010.

Animals and other organisms

Policy information about studies involving animals; ARRIVE guidelines recommended for reporting animal research

Laboratory animals	C. elegans strains: N2, SP486, strains with mes-2(ax2059[mes-2::GFP]) and him-8(e1489), as well as multiple strains which production is described in the manuscript. Male and hermaphrodites were used at 20-24 hours post L4.

Wild animals	No wild animals were used in the study.
Field-collected samples	No field collected samples were used in the study.
Ethics oversight	Hebrew University of Jerusalem

Note that full information on the approval of the study protocol must also be provided in the manuscript.
